# Exercise and visual training interventions for myopia in children and adolescents: a systematic review with a focus on near–far accommodation

**DOI:** 10.3389/fpubh.2026.1889393

**Published:** 2026-08-12

**Authors:** Xixi Ge, Dongxu Gao, Ying Fang, Sijun Wu, Enda Zhen, Chuifeng Kong, Yujun Cai

**Affiliations:** 1 School of Physical Education, Shanghai University of Sport, Shanghai, China; 2 Division of Sports Science and Physical Education, Tsinghua University, Beijing, China; 3 Department of Performing Arts, Rizhao Marine Engineering Vocational College, Rizhao, Shandong, China; 4 Mengcun Hui Autonomous County People’s Court, Cangzhou, China

**Keywords:** accommodative function, children and adolescents, exercise intervention, myopia, near–far accommodative training, visual training

## Abstract

**Objective:**

This systematic review examined the effects of near–far accommodation-based exercise and visual training on myopia-related outcomes in children and adolescents, with the aim of informing non-pharmacological and behavioral approaches to myopia prevention and control.

**Background:**

Myopia in children and adolescents is a major public health concern and may increase the risk of high myopia and related ocular complications. In recent years, interventions such as alternating near–far fixation, accommodative facility training, visual tracking, and virtual reality–based visual training have received increasing attention. However, existing studies vary substantially in intervention modalities, outcome measures, and follow-up periods, and the effectiveness of these interventions for myopia-related outcomes remains unclear.

**Methods:**

In May 2026, a comprehensive search was conducted across five electronic databases, including Embase, PubMed, Cochrane, Web of Science, and CNKI, as well as Google Scholar. According to study design, the risk of bias in randomized controlled trials was assessed using the RoB 2 tool, whereas the risk of bias in non-randomized intervention studies was evaluated using the ROBINS-I tool. This review included only experimental studies that examined the effects of near–far accommodation-based exercise and visual training on myopia-related indicators in children and adolescents.

**Results:**

A total of 13 articles met all inclusion criteria and were included in this systematic review. The evidence suggested that near–far accommodation-based visual training or sports vision interventions may help slow axial elongation or reduce the myopic shift in spherical equivalent refraction (SER/SE). Improvements in UCVA, KVA, and AF were also observed across multiple studies.

**Conclusion:**

Near–far accommodation-based exercise and visual training may improve certain functional vision and accommodative outcomes in children and adolescents. However, their effects on myopia prevention and control should be interpreted cautiously. Axial length and cycloplegic spherical equivalent refraction are the core outcomes for evaluating myopia progression, but current evidence for these outcomes remains limited. Therefore, no confirmatory conclusion can yet be drawn regarding consistent myopia-control effects. Future well-designed randomized controlled trials with adequate follow-up and complete outcome reporting are needed.

**Systematic review registration:**

https://www.crd.york.ac.uk/PROSPERO/view/CRD420261390075, registration number: CRD420261390075.

## 1 Introduction

Myopia is a type of refractive error in which, when ocular accommodation is relaxed, light rays entering the eye parallel to the visual axis are refracted by the ocular optical system and focused in front of the retina, resulting in blurred distance vision (1–3). At present, myopia has become a major public health concern worldwide. The global number of individuals with myopia is projected to increase from approximately 1.406 billion in 2000 to approximately 4.758 billion by 2050, accounting for 49.8% of the global population. During the same period, the number of individuals with high myopia is expected to rise to approximately 938 million, representing 9.8% of the global population (4). Among all population groups, children and adolescents are a key population in whom myopia onset and progression occur most rapidly. It is estimated that by 2050, the pooled prevalence of myopia among children and adolescents will reach 39.80%, affecting more than 740 million individuals. The trends toward earlier onset and accelerated progression of myopia therefore warrant particular attention (5). Myopia not only impairs distance visual clarity and the quality of daily learning and life among children and adolescents, but also increases the risk of ocular complications associated with high myopia, including myopia-related fundus lesions, retinal detachment, and glaucoma, thereby imposing a long-term burden on individual visual health and healthcare systems (6, 7). Therefore, exploring safe, sustainable, and age-appropriate strategies for myopia prevention and control in children and adolescents is of considerable practical significance.

The onset and progression of myopia are not merely attributable to refractive abnormalities, but rather result from the combined effects of genetic, environmental, and vision-related behavioral factors. Previous studies have shown that prolonged near work, insufficient outdoor activity, longer duration of education, and increased sustained visual load are closely associated with the onset and progression of myopia in children and adolescents (8, 9). Among these factors, accommodative function is considered to play a key role in linking the external visual environment with changes in ocular growth. Abnormalities in accommodative function, such as accommodative lag, may generate persistent hyperopic defocus signals during near fixation, thereby influencing the regulation of ocular growth and potentially contributing to axial elongation and myopia progression (10–14). Accordingly, accommodative function is not only an important component of the visual system, but may also represent a key indicator in interventions for myopia prevention and control and may serve as an important mediating pathway through which physical activity influences myopia.

In recent years, visual training targeting accommodative function, sports vision training, and exercise-embedded near–far accommodative training have attracted increasing attention. Near–far accommodative training repeatedly stimulates contraction and relaxation of the ciliary muscle by alternating fixation distances, presenting dynamic visual targets, or guiding repeated shifts of gaze between near and distant targets. This process may help improve the speed and stability of accommodative responses and thereby enhance overall visual function (15–17). Accordingly, VR/AR-based visual training, naked-eye three-dimensional visual training, ciliary muscle training devices, and ocular exercise programs can be classified as direct accommodative or visual training methods. By contrast, exercise-based interventions should be considered exercise-embedded near–far accommodative training only when their protocols explicitly incorporate near–far target recognition, dynamic visual tracking, rapid fixation shifts, or visual refocusing tasks. This classification is consistent with the categorization approach adopted in existing reviews in the field of visual training (18). Physical activities, particularly ball games, orienteering, and activities involving visual tracking tasks, often require changes in target distance, rapid gaze shifts, dynamic tracking, and spatial judgment. These visual demands can generate visual stimulation patterns similar to near–far accommodative switching within sports contexts (19). In terms of specific sports, ball games such as football and table tennis require children to continuously track the trajectory of the ball and judge spatial distance. Some studies have further transformed the exercise process into near–far target recognition tasks by placing visual markers, such as numbers or letters, on balls or sports equipment, thereby strengthening ciliary muscle accommodative training (20–23). Orienteering (24) involves repeated switching between map recognition and real-world environmental search, thereby creating near–far visual transitions accompanied by ocular pursuit, saccades, fixation, focusing, and alternating visual tracking. These processes may enhance the regulatory capacity of the extraocular muscles and ciliary muscle. Cheerleading (25), as a closed-skill sport, artificially increases changes in visual targets and target distance through pom–pom tossing and catching, hand-position changes, and training strategies such as “eyes following the hands” and “clearly identifying the texture of the pom-poms.” These elements create a training context involving both visual tracking and accommodative regulation. In addition, some studies have suggested that near–far accommodative training may not only improve accommodative function, but may also further influence clinical myopia-related indicators, such as axial length and refractive status, as well as functional myopia-related indicators, including uncorrected visual acuity and kinetic visual acuity (26–28).

Childhood and adolescence are critical periods for visual system development and are also the stages during which myopia onset and progression occur most rapidly. During this period, the visual system exhibits a relatively high degree of plasticity, which may make training interventions targeting accommodative function more likely to produce meaningful effects (15–17). Therefore, evaluating the potential effects of various near–far accommodative training approaches on myopia prevention and control in children and adolescents may provide important insights. Existing reviews on exercise- or vision-based interventions for myopia have mainly focused on outdoor activities, different exercise modalities, eye exercises, and emerging digital visual training approaches. These reviews have provided preliminary evidence suggesting that increasing outdoor time, engaging in regular physical activity, or undertaking certain forms of visual training may have beneficial effects on myopia-related indicators in children and adolescents. However, several limitations remain in the existing literature. Most reviews have evaluated outdoor exposure, general physical activity, or ocular exercises as broad intervention categories, with limited further attention to the effects of key visual components, such as near–far fixation shifts, dynamic visual-target tracking, and ciliary-muscle accommodative regulation, on myopia-related outcomes in children and adolescents. Moreover, the relationships between these components and clinical myopia-related indicators, such as axial length and refractive status, as well as functional myopia-related indicators, including uncorrected visual acuity and kinetic visual acuity, have not been sufficiently synthesized. Therefore, it is necessary to systematically review relevant studies on visual training and physical activity interventions by focusing on the core feature of near–far accommodative training, in order to clarify its effects on myopia-related outcome indicators in children and adolescents.

## 2 Materials and methods

### 2.1 Protocol and registration

This systematic review was reported in accordance with the Preferred Reporting Items for Systematic Reviews and Meta-Analyses (PRISMA) guidelines (6, 29, 30). The review protocol was registered with the International Prospective Register of Systematic Reviews (PROSPERO), with the registration number CRD420261390075.

### 2.2 Search strategy

To ensure the comprehensiveness of the literature review, three researchers independently searched multiple electronic databases, including Web of Science, PubMed, Embase, Cochrane, and CNKI. The search covered the period from database inception to May 1, 2026. Keyword combinations were used in each database, including terms related to myopia and visual outcomes, such as “myopia,” “refractive error,” and “visual acuity,” as well as terms related to intervention approaches, such as “near–far accommodative training,” “ciliary muscle training,” and “visual training.” Boolean operators were used to combine search terms: “OR” was applied to expand terms within the same concept, whereas “AND” was used to combine different concepts, thereby improving both the comprehensiveness and specificity of the search strategy. Detailed search strategies are provided in the Supplementary materials.

In addition, Google Scholar was used as a supplementary source to complement the electronic database search and reduce the likelihood of missing relevant studies. The Google Scholar search used free-text combinations related to the research topic, including “myopia,” “accommodation,” “accommodative lag,” “accommodative response,” and “near work.” Considering the large number of Google Scholar results and their relevance-based ranking, only the first 200 records ranked by relevance were screened. To further ensure the comprehensiveness of the literature retrieval, the reference lists of the included studies and relevant systematic reviews and meta-analyses were manually searched, and eligible studies were included in the present review. The Google Scholar records were merged with records retrieved from other databases, followed by duplicate removal and screening. The supplementary Google Scholar search did not identify any additional studies that met the inclusion criteria; the retrieved records were mainly duplicates of studies identified from other databases or studies that did not meet the eligibility criteria.

### 2.3 Eligibility criteria

This study followed the Preferred Reporting Items for Systematic Reviews and Meta-Analyses (PRISMA) guidelines and systematically evaluated the effects of near–far accommodative training on visual health outcomes (29, 30). The inclusion and exclusion criteria were developed according to the PICOS framework specified in PRISMA, covering population (P), intervention (I), comparator (C), outcomes (O), and study design (S) (Table 1). Studies were included if they met the following criteria: (1) the participants were school-aged children and adolescents aged 6–18 years, regardless of sex, including non-myopic individuals, individuals with myopia, or individuals at risk of myopia. According to the International Myopia Institute (IMI), myopia is commonly defined in clinical and epidemiological studies as a spherical equivalent refraction (SER/SE) of ≤ −0.50 D; non-myopic individuals are generally defined as those with SER/SE > −0.50 D; high myopia is usually defined as SER/SE ≤ −6.00 D; and individuals at risk of myopia, or those with pre-myopia, are characterized by SER/SE > −0.50 D and SER/SE ≤ +0.75 D. (2) The intervention involved any form of accommodative visual training, near–far accommodative training, or accommodation-related visual intervention embedded in physical activity. Specifically, studies were included only when the intervention protocol contained at least one clearly identifiable visual component related to near–far accommodation. These components included: (a) repeated fixation shifts between near and distant targets; (b) visual stimuli presented at different depths or simulated viewing distances; (c) dynamic targets requiring accommodative focusing, refocusing, or visual tracking; and (d) physical activities that explicitly incorporated visual recognition, fixation shifts, or cross-distance target-tracking tasks. (3) The study included a comparator group receiving no intervention, routine activities, regular physical education classes, health education, or other control conditions that did not involve visual accommodative training. (4) The study reported at least one myopia-related outcome, including visual performance, ocular structural or refractive indicators, accommodative function indicators, or other visual health outcomes. (5) The study employed a randomized controlled trial or non-randomized controlled trial design.

**Table 1:** Inclusion criteria based on the PICOS framework.

PICOS	Details
Population	School-aged children and adolescents, both male and female, aged 6–18 years.
Intervention	Any form of accommodative visual training, near–far accommodative training, or accommodation-related visual intervention embedded in physical activity.
Comparator	Regular study routines, daily activities, regular physical education or ordinary sports activities, or no-intervention control.
Outcomes	Visual performance indicators, including uncorrected visual acuity, binocular visual acuity, results measured separately for the right and left eyes, and kinetic visual acuity; ocular structural or refractive indicators, including axial length, spherical equivalent refraction, refractive error, and corneal curvature; accommodative function indicators, including accommodative lag, accommodative facility, accommodative amplitude, and accommodative accuracy; and other visual health-related indicators.
Study design	Randomized controlled trials and non-randomized interventional studies were included, including quasi-experimental studies in which intervention and control groups were assigned by the investigators. Studies were required to include a comparable control group and to measure the primary outcomes consistently before and after the intervention. Single-group pre–post studies without a control group were not included in the primary evidence synthesis.

Studies were excluded if they met any of the following criteria: (1) the full text was unavailable; (2) the study population was outside the age range of 6–18 years, or included individuals with clearly diagnosed pathological ocular diseases; (3) the intervention was unrelated to near–far accommodative training, accommodative visual training, or visual accommodative stimuli embedded in physical activity. General physical activity interventions were not directly included unless the intervention protocol explicitly described task components related to accommodation, near–far fixation shifts, or dynamic visual tracking; (4) the study design was observational, cross-sectional, a review, conference abstract, case report, letter to the editor, or short communication; (5) the study did not report extractable outcomes related to myopia or visual health; (6) the intervention was a multicomponent intervention, but the independent effects of near–far accommodative training or accommodation-related visual intervention could not be distinguished; or (7) the article was published in a language other than Chinese or English.

### 2.4 Study selection

**Figure 1:**
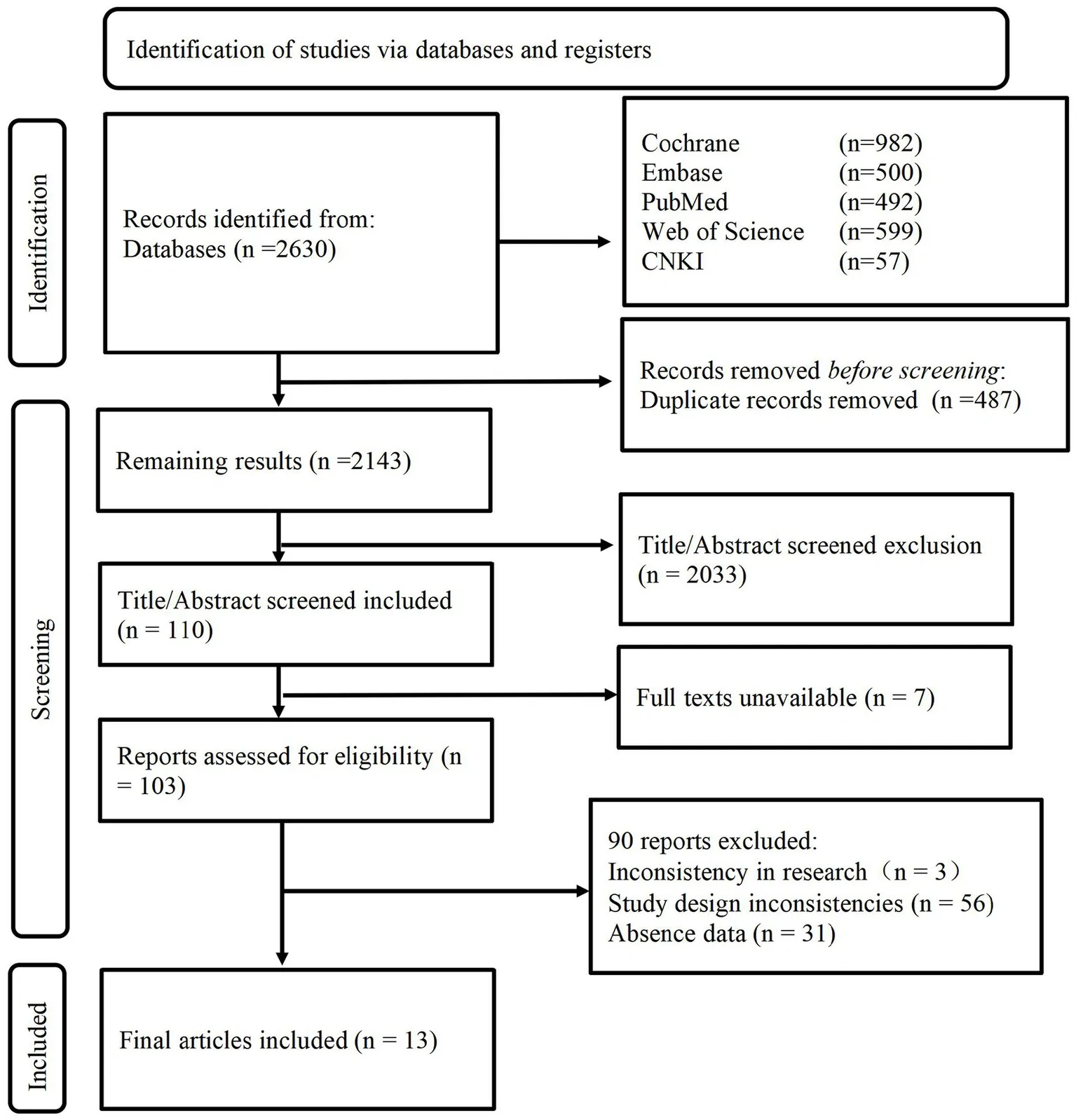
PRISMA flow diagram of the study selection process.

After searches were conducted in the five electronic databases, the retrieved records, including titles, authors, and publication years, were imported into Zotero reference management software for duplicate removal. Two independent reviewers (Ge and Fang) screened the titles and abstracts to identify potentially eligible studies for full-text review. Subsequently, the same reviewers assessed the full texts of all relevant studies according to the predefined inclusion and exclusion criteria and determined their eligibility for inclusion in the review. Throughout the study selection process, the two reviewers (Ge and Fang) worked independently. Any disagreements were resolved through consultation with a third reviewer (Gao) until consensus was reached. Details of the study selection process are presented in Figure 1.

### 2.5 Data extraction, risk-of-bias assessment, and evidence synthesis

After completion of the study selection process, two independent reviewers (Ge and Fang) extracted relevant data from each included study, including the study title, population characteristics, study design, intervention characteristics, training type, outcome measures, and main findings.

In this review, risk of bias was assessed using tools appropriate to the design of the included studies. For individually randomized controlled trials, the revised Cochrane risk-of-bias tool for randomized trials (Risk of Bias 2, RoB 2) was used. For cluster-randomized controlled trials, the RoB 2 extension for cluster-randomized trials (RoB 2 for cluster-randomized trials, RoB 2 CRT) was applied (31). For non-randomized comparative intervention studies, the Risk Of Bias In Non-randomized Studies of Interventions (ROBINS-I) tool was used (32).

The RoB 2 tool assesses risk of bias in randomized controlled trials across five domains: bias arising from the randomization process, bias due to deviations from the intended interventions, bias due to missing outcome data, bias in measurement of the outcome, and bias in selection of the reported result. The risk of bias in each domain was judged as “low risk of bias,” “some concerns,” or “high risk of bias,” and an overall risk-of-bias judgment was then derived based on the domain-level assessments. For cluster-randomized controlled trials, RoB 2 CRT further considers issues specific to cluster-randomized designs, including the cluster randomization process, the timing of participant identification or recruitment, handling of clustering effects, and whether the analytical approach was appropriate for the study design.

The ROBINS-I tool was used to assess risk of bias in non-randomized intervention studies. This tool evaluates seven domains: bias due to confounding, bias in selection of participants into the study, bias in classification of interventions, bias due to deviations from the intended interventions, bias due to missing data, bias in measurement of outcomes, and bias in selection of the reported result. The risk of bias in each domain was judged as “low,” “moderate,” “serious,” or “critical,” and an overall risk-of-bias judgment was determined accordingly.

This review adopted a structured narrative synthesis rather than a quantitative meta-analysis. Before determining the approach to evidence synthesis, we re-examined the extracted data and assessed the feasibility of pooling effect estimates. The results showed substantial heterogeneity across the included studies in terms of study population, intervention type, intervention duration, training dose, outcome measures, measurement methods, and statistical reporting formats. In addition, some studies did not provide key data required for effect-size pooling, such as standard deviations, confidence intervals, or comparable post-intervention results. Therefore, conducting a quantitative synthesis under these conditions could have produced misleading results. Because no pooled effect estimates were generated in this review, statistical heterogeneity testing, sensitivity analysis, and publication bias assessment were not further performed. Instead, we conducted a structured narrative synthesis according to the major outcome domains and qualitatively compared differences across studies to present the current evidence more accurately.

## 3 Results

### 3.1 Study selection

A total of 2,630 relevant records were identified through database searches and other supplementary sources. After duplicate removal, 2,143 records remained. Following title and abstract screening, 2,033 records that were clearly ineligible were excluded. A further seven records were excluded because the full text could not be obtained, leaving 103 full-text articles for eligibility assessment. After further full-text review, 13 studies were ultimately included according to the predefined inclusion and exclusion criteria regarding population, intervention type, comparator, outcomes, and study design. The study selection process is presented in the PRISMA flow diagram.

The included studies were published between 2008 and 2026. The study populations mainly consisted of children and adolescents, with most participants being school-aged children or adolescents. The intervention types primarily included various forms of visual training and exercise interventions incorporating near–far accommodative training. Among these, studies on visual training interventions were relatively more common, followed by exercise-based interventions. The intervention duration and training dose varied considerably, ranging from immediate or several-week interventions to longer interventions lasting 3–6 months, with some studies extending up to 2 years. The comparator conditions mainly included no visual training, maintenance of usual lifestyle, regular physical education, or other routine management measures (Table 2).

**Table 2:** Characteristics of included studies.

Study	Sample (T/C)	Age	Intervention (T/C)	Duration (weeks)	Frequency (sessions/week)	Session duration (min/session)	Outcome measures	Main findings
Xu et al. (28)	Students (30/30)	T: 10.53 ± 1.57; C: 10.67 ± 1.30	SVS + VR-based visual training / SVS	12	7	20	UDVA, NVA, AL, mCT	AL: T baseline, 24.66 ± 0.64 mm; 3-month *Δ*, 0.063 ± 0.060 mm, between-group *p* = 0.037; 6-month *Δ*, 0.166 ± 0.109 mm, *p* = 0.172. mCT: T baseline, 290.77 ± 48.25 μm; 3-month *Δ*, +22.63 ± 36.17 μm, *p* = 0.005; 6-month *p* = 0.145. UDVA and NVA improved at 3 months (*p* = 0.000 and *p* = 0.034, respectively).
Xie et al. (27)	Students (131/132)	6–18 years; T: 11.04 ± 1.96; C: 10.58 ± 1.96	Naked-eye three-dimensional vision training/normal daily life	24	7	20	AL, SER	AL: T baseline, 24.35 ± 0.83 mm; 6-month *Δ*, 0.176 mm (95% CI: 0.155 to 0.196); between-group difference, −0.056 mm (95% CI: −0.085 to −0.027). SER: T baseline, −2.08 (1.12) D; 6-month *Δ*, −0.251 D (95% CI: −0.311 to −0.191).
Zheng et al. (36)	Students (26/27)	6–18 years	Eye-protection exercises + table tennis/regular study and daily life	24	7; 3	20; 45	UDVA-L, UDVA-R, BVA	UDVA-L: 0.565 ± 0.042 to 0.912 ± 0.041 at 3 months (*Δ* +0.347); 0.908 ± 0.042 at 6 months (*Δ* +0.343), both *p* < 0.01. UDVA-R: 0.539 ± 0.042 to 0.881 ± 0.047 at 3 months (*Δ* +0.342); 0.877 ± 0.046 at 6 months (*Δ* +0.338), both *p* < 0.01. BVA: 0.704 ± 0.048 to 1.062 ± 0.046 at 3 months; 1.054 ± 0.045 at 6 months, both *p* < 0.01.
Yin et al. (22)	Students (44/40/43/33)	10–11 years	Physical activity-embedded near–far accommodative training (15-, 30-, and 60-session groups)/regular physical activity	16	3	40	KVA, UDVA	KVA: 30-session group, 0.268 ± 0.214 to 0.486 ± 0.327 (*Δ* +0.218); 60-session group, 0.333 ± 0.304 to 0.424 ± 0.324 (*Δ* +0.091); 15-session group, *p* > 0.05. UDVA: 30-session group, 4.775 ± 0.306 to 4.937 ± 0.269 (*Δ* +0.162, *p* < 0.05); 60-session group, 4.807 ± 0.335 to 4.958 ± 0.280 (*Δ* +0.151, *p* < 0.05).
Zhou et al. (23)	Students (39/38/38/38)	10–11 years	Ciliary muscle training embedded in PE class with different visual target presentation durations (1 s/3 s/5 s)/same physical activity	32	3	40	KVA, UDVA, AL, AF	In the 1-s, 3-s, and 5-s groups, KVA increased by +0.10, +0.12, and +0.09, respectively; UDVA increased by +0.11, +0.14, and +0.11, respectively; AL increased by +0.20, +0.20, and +0.22 mm, respectively; and AF increased by +0.22, +0.26, and +0.16 cpm, respectively.
Han et al. (20)	Students (75/127)	9.5 years	Regular classes + tennis lessons/extracurricular activities and regular PE class	40	2; 4	40; 30	UDVA-L, UDVA-R, detection rate of poor vision	Boys’ UDVA-L/R: T baseline, 5.06 ± 0.16/5.08 ± 0.11; 10 months, 5.00 ± 0.13/5.00 ± 0.12 (*Δ* −0.06/−0.08). In the third assessment, T was higher than C (*p* = 0.02). Girls’ UDVA-L/R: T baseline, 5.05 ± 0.16/5.05 ± 0.15; 10 months, 4.97 ± 0.13/4.97 ± 0.14 (*Δ* −0.08/−0.08). In the third assessment, T was higher than C (*p* < 0.01). The detection rate of poor vision was lower than that in the control group (*p* < 0.01).
Lin et al. (21)	Students (12/12/12)	Table tennis: 7.4 ± 1.43; badminton: 7.9 ± 1.10; C: 8.20 ± 1.75	Table tennis or badminton/regular PE games	24	3	90	SER, AL, AF	Table tennis group: left/right SER *Δ* −0.18/−0.10 D (both *p* > 0.05); left/right AL *Δ* +0.54/+0.65 mm (both *p* < 0.05); AF improved significantly in both eyes (*p* < 0.05). Badminton group: left-eye SER *Δ* −0.04 D (*p* < 0.05), right-eye SER *Δ* −0.15 D (*p* > 0.05); left/right AL *Δ* +0.68/+0.52 mm (both *p* < 0.05).
Wang et al. (24)	Students (60/60)	10–11 years	Orienteering visual training/regular study and daily life	10	3	45	UDVA, SE	UDVA: 4.66 ± 0.05 to 4.73 ± 0.64 (*Δ* +0.07, *p* < 0.01). SE: −3.35 ± 0.92 to −2.99 ± 0.25 D (*Δ* +0.36, *p* < 0.01).
Xie et al. (34)	Students (53/53)	T: 10.61 ± 1.77; C: 10.83 ± 1.69	Near-use convex-lens prism + physiological visual eye exercises/near-use convex-lens prism	104	14	3	SER, AL	SER: −1.17 ± 0.47 to −2.05 ± 0.81 D at 2 years (*Δ* −0.81 D, *p* < 0.05; between-group *p* = 0.0001). AL: 23.91 ± 0.66 to 24.21 ± 0.36 mm (*Δ* +0.23 mm, *p* < 0.05; between-group *p* = 0.023).
Zhao et al. (35)	Students (39/37)	T: 10.4; C: 10.3	SU-001 ciliary muscle training/no training	16	14	15	UDVA, SER	UDVA: 0.71 ± 0.36 to 1.20 ± 0.56 at 4 months (*Δ* +0.49, *p* < 0.01).
Jin et al. (25)	Students (39/32; no blank control group)	NR; Grade 4 students	Cheerleading-based dynamic visual acuity training/football-based dynamic visual acuity training	16	3	40	KVA, UDVA	Cheerleading group: KVA, 0.338 ± 0.240 to 0.497 ± 0.249 (*Δ* +0.159); UDVA, 4.526 ± 0.245 to 4.741 ± 0.241 (*Δ* +0.215). Football group: KVA, 0.315 ± 0.228 to 0.549 ± 0.203 (*Δ* +0.234); UDVA, 4.491 ± 0.135 to 4.600 ± 0.134 (*Δ* +0.109).
Allen et al. (15)	Myopic participants (*n* = 93)	16.7 ± 2.1 years; only participants younger than 18 years were considered	Spherical-aberration-modified contact lenses and/or visual training/control contact lenses and/or no training	6	7	18	LoA, DAF, NAF	LoA decreased in the contact lens treatment group (*F* = 11.23, *p* = 0.001), whereas it increased in the contact lens control group (*p* = 0.029). No significant effect was observed in the visual training group. NAF: VT treatment group, 5.19 ± 2.33 to 7.27 ± 2.05 cpm (*Δ* +2.08, *p* < 0.001). DAF improved in both groups, but no between-group difference was observed (*p* = 0.158).
Hu et al. (33)	Students (11/9)	T: 9.36 ± 1.75; C: 8.44 ± 1.81	AR-based visual training/rest	Single session	1	30	ChT, AF, SER	ChT in the 0–1 mm zone: 332.85 ± 79.00 to 324.11 ± 73.52 μm (*Δ* −8.74 μm, 95% CI: −13.81 to −3.68, *p* = 0.007). ChT in the 1–3 mm zone: 327.30 ± 74.54 to 318.60 ± 68.60 μm (*p* = 0.018). Monocular AF: AR group, 5.05 ± 1.38 to 8.05 ± 1.81 cpm (*Δ* +3.00, *p* = 0.004); no significant change was observed in the control group (*p* = 0.367). Binocular AF showed no significant change (*p* = 0.127).

*Δ* represents the change value. T, treatment/intervention group; C, control group; SD, standard deviation; CI, confidence interval; MD, mean difference; *p*, probability value; D, diopter; cpm, cycles per minute; SVS, single-vision spectacles; VR, virtual reality; AR, augmented reality; UDVA, uncorrected distance visual acuity; NVA, near visual acuity; BVA, binocular visual acuity; KVA, kinetic visual acuity; AL, axial length; SER, spherical equivalent refraction; mCT, macular choroidal thickness; ChT, choroidal thickness; CC, corneal curvature; AF, accommodative facility; AMP, amplitude of accommodation; LoA, lag of accommodation; DAF, distance accommodative facility; NAF, near accommodative facility; PRA, positive relative accommodation; NRA, negative relative accommodation.

### 3.2 Risk-of-bias assessment

**Figure 2:**
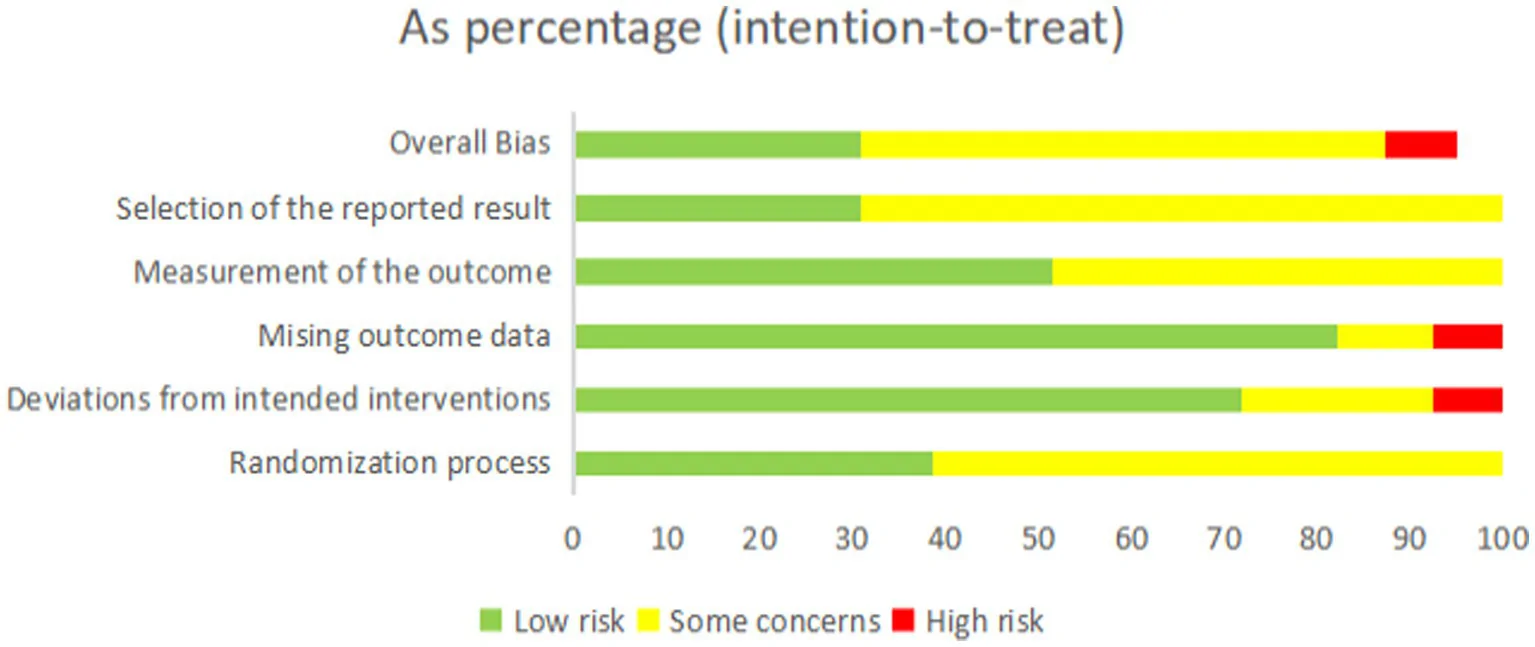
Risk-of-bias plot. This figure presents only the RoB 2 assessment results for the 11 individually randomized controlled trials; the results for the cluster-randomized study and the non-randomized study are presented in Table 3.

A total of 13 included studies were assessed for risk of bias in this review. Among them, 11 individually randomized controlled trials were assessed using the RoB 2 tool, one cluster-randomized study was assessed using the RoB 2 tool for cluster-randomized trials, and one non-randomized comparative intervention study was assessed using ROBINS-I V2. The overall assessment results are presented in Table 3, and the RoB 2 visualization for the 11 individually randomized controlled trials is shown in Figure 2. Because ROBINS-I, the standard RoB 2 tool, and the RoB 2 tool for cluster-randomized trials differ in their assessment domains and judgment levels, the non-randomized study and the cluster-randomized study were not combined into the automatically generated visualization for standard RoB 2 assessments. Instead, their assessment tools, overall judgments, and main sources of bias are reported separately in the summary table.

**Table 3:** Risk-of-bias assessment.

No.	Study	Study design	Assessment tool	Outcomes assessed	Overall risk of bias
1	Xu et al. (28)	Individually randomized controlled trial (RCT)	RoB 2 (individual randomized parallel-group trial)	AL, mCT, UCVA, NVA	Some concerns
2	Xie et al. (27)	Individually randomized controlled trial (RCT)	RoB 2 (individual randomized parallel-group trial)	AL, SER, AE	Low
3	Zheng et al. (36)	Individually randomized controlled trial (RCT)	RoB 2 (individual randomized parallel-group trial)	UCVA (left eye, right eye, and binocular)	High
4	Yin et al. (22)	Individually randomized controlled trial (RCT)	RoB 2 (individual randomized parallel-group trial)	KVA, UDVA	Low
5	Zhou et al. (23)	Individually randomized controlled trial (RCT)	RoB 2 (individual randomized parallel-group trial)	KVA, UDVA, AL, AF	Some concerns
6	Han et al. (20)	Individually randomized controlled trial (RCT)	RoB 2 (individual randomized parallel-group trial)	UCVA (left and right eyes), detection rate of poor vision	Some concerns
7	Lin et al. (21)	Individually randomized controlled trial (RCT)	RoB 2 (individual randomized parallel-group trial)	SER/SE, AL, K, AL/CR, AMP, NRA, PRA, AF	Some concerns
8	Wang et al. (24)	Individually randomized controlled trial (RCT)	RoB 2 (individual randomized parallel-group trial)	UCVA, refractive error, visual fatigue	Some concerns
9	Xie et al. (34)	Individually randomized controlled trial (RCT)	RoB 2 (individual randomized parallel-group trial)	Myopic refractive error/SE, AL	Low
10	Allen et al. (15)	Individually randomized controlled trial (RCT)	RoB 2 (individual randomized parallel-group trial)	LoA, distance AF, near AF, PRT, NRT	Some concerns
11	Hu et al. (33)	Individually randomized controlled trial (RCT)	RoB 2 (individual randomized parallel-group trial)	ChT (0–1 mm and 1–3 mm), monocular AF	Some concerns
12	Zhao et al. (35)	Cluster-randomized controlled trial (class-level randomization)	RoB 2 CRT	Change in cycloplegic refractive classification after 4 months; change in UCVA after 4 months	High
13	Jin et al. (25)	Non-randomized comparative intervention study	ROBINS-I V2	Change in UCVA after 16 weeks, KVA	Serious

Individually randomized controlled trials were assessed using RoB 2; the cluster-randomized study was assessed using RoB 2 CRT; and the non-randomized comparative intervention study was assessed using ROBINS-I V2. The assessment domains and risk judgment levels are not fully identical across these tools.

Among the 11 individually randomized controlled trials, three studies were judged to have a low overall risk of bias, seven raised some concerns, and one was judged to have a high risk of bias. The main sources of bias included insufficient reporting of the randomization process or allocation concealment, lack of pre-registration or statistical analysis plans, potential influence of knowledge of the intervention on outcome measurement for some outcomes, and inadequate handling of missing data in some studies. In addition, one cluster-randomized study was judged to have a high overall risk of bias, mainly because randomization was conducted at the class level but clustering effects were not adequately considered in the analysis. One non-randomized comparative intervention study was judged to have a serious overall risk of bias, mainly because of non-random allocation and insufficient control for important confounding factors (see Table 4).

**Table 4:** Full-text excluded studies and reasons for exclusion.

Study	Reason for exclusion
He et al. (51)	Did not meet review criteria as the intervention was outdoor time/light exposure rather than near-far accommodative or visual training.
Li et al. (52)	Did not meet review criteria as the intervention focused on outdoor exposure rather than near-far accommodative training.
Ying et al. (53)	Not eligible as this was an umbrella review of systematic reviews and meta-analyses.
Yu et al. (54)	Not eligible as this was a systematic review and meta-analysis of risk factors.
Morgan et al. (55)	Not eligible as this was a narrative review on myopia aetiology and prevention.
Dhakal et al. (56)	Not eligible as this was an overview of systematic reviews.
Xiong et al. (57)	Not eligible as this was a systematic review and meta-analysis of outdoor activities.
Cheng and Zhang (58)	Not eligible as this was a systematic review and network meta-analysis.
Li et al. (49)	Not eligible as this was a narrative review.
Xie et al. (59)	Not eligible as this was a cross-sectional prediction-model study.
Zhang et al. (60)	Not eligible as this was a cross-sectional survey on myopia prevalence and influencing factors.
Xu et al. (61)	Not eligible as this was a cross-sectional survey on myopia status and influencing factors.
Wang et al. (42)	Did not meet review criteria as participants were patients with primary open-angle glaucoma.
Wang et al. (42)	Not eligible as this was an observational study without an intervention.
Read et al. (62)	Not eligible as this was a longitudinal observational study of light exposure.
Fan et al. (63)	Did not meet review criteria as the intervention was a general badminton exercise prescription without clearly defined near-far accommodative training.
Wu (64)	Not eligible as this was a master’s thesis rather than a peer-reviewed journal article.
Pan (123)	Not eligible as this was a master’s thesis rather than a peer-reviewed journal article.
Zhang (65)	Not eligible as this was a master’s thesis rather than a peer-reviewed journal article.
Nie (66)	Not eligible as this was a master’s thesis rather than a peer-reviewed journal article.
Zhou (67)	Not eligible as this was a master’s thesis rather than a peer-reviewed journal article.
Yan (68)	Not eligible as this was a master’s thesis rather than a peer-reviewed journal article.
Chen (69)	Not eligible as this was a master’s thesis rather than a peer-reviewed journal article.
Xue et al. (70)	Not eligible as this was a cross-sectional study on gait kinematics, not an intervention study.
Huang (71)	Not eligible as this was a master’s thesis rather than a peer-reviewed journal article.
Xie (72)	Not eligible as this was a master’s thesis rather than a peer-reviewed journal article.
Song et al. (73)	Not eligible as this was a comparative observational study rather than a controlled intervention study.
Chen et al. (74)	Not eligible as this was a cross-sectional study on dynamic visual acuity and table-tennis training experience.
Jouira et al. (75)	Did not meet review criteria as the outcome was postural balance rather than myopia-related visual function.
Liu et al. (19)	Not eligible as this was a network meta-analysis, not a primary intervention study.
Tao et al. (76)	Not eligible as this was an expert recommendation rather than a primary intervention study.
Chen et al. (77)	Not eligible as this was a prospective cohort study rather than an intervention study.
Lundberg et al. (78)	Not eligible as this was an observational cohort study without an intervention.
Li et al. (79)	Did not meet review criteria as the intervention was acute physical exercise rather than near-far accommodative or visual training.
Mei et al. (80)	Not eligible as this was a systematic review and meta-analysis.
Al-Mohtaseb et al. (81)	Not eligible as this was a review on dry eye disease and digital screen use.
Xue et al. (70)	Not eligible as this was a cross-sectional study on sleep–wake schedules and self-reported myopia.
Wang et al. (82)	Did not meet review criteria as participants were medical students and the outcome was visual fatigue rather than myopia-related visual function.
Yang et al. (83)	Not eligible as this was a cross-sectional correlation study on physical activity and myopia.
O’Donoghue et al. (84)	Not eligible as this was an observational risk-factor study rather than an intervention study.
Liao et al. (85)	Did not meet review criteria as the intervention was outdoor illumination and general exercise rather than near-far accommodative or visual training.
Ma et al. (86)	Not eligible as this was a cross-sectional study on physical activity and myopia prevalence.
Lu et al. (87)	Did not meet review criteria as the outcome was short-term eye strain relief rather than myopia-related visual function.
Biswas et al. (88)	Not eligible as this was a narrative review on environmental and lifestyle factors related to myopia.
Zhang et al. (89)	Did not meet review criteria as the outcome focused on physical and mental health rather than myopia-related visual function.
Ren and Lu (90)	Not eligible as this was a theoretical study on visual training curriculum rather than a primary intervention study.
Ren and Yang (91)	Did not meet review criteria as visual training was combined with orthokeratology and cloud-clip monitoring.
Wang et al. (92)	Did not meet review criteria as the intervention was optical lens treatment rather than near-far accommodative or visual training.
Gao and Ye (93)	Did not meet review criteria as visual training was combined with orthokeratology and repeated low-energy red-light therapy.
Liu et al. (94)	Not eligible as this was a review article rather than a primary intervention study.
Qiao et al. (95)	Not eligible as this was a theoretical article on acupuncture rather than a primary intervention study.
Wang (96)	Not eligible as this was a master’s thesis rather than a peer-reviewed journal article.
Peng (97)	Not eligible as this was a master’s thesis rather than a peer-reviewed journal article.
Wang et al. (98)	Not eligible as this was an observational study of accommodative function rather than an intervention study.
Yuda et al. (99)	Did not meet review criteria as no eligible comparable control group was included.
Chen et al. (100)	Did not meet review criteria as participants had convergence insufficiency with accommodative dysfunction rather than myopia-related outcomes.
Shyu et al. (101)	Did not meet review criteria as this was an immediate-effect pilot study in youth badminton athletes.
Scheiman et al. (102)	Not eligible as this was a network meta-analysis rather than a primary intervention study.
Yu et al. (103)	Not eligible as this was a narrative review on visual function and myopia progression.
Wang et al. (96)	Not eligible as this was a narrative review on myopia and accommodative function.
Zhang et al. (104)	Not eligible as this was a narrative review on the relationship between myopia and accommodation.
Walline et al. (105)	Not eligible as this was a systematic review rather than a primary intervention study.
Jing et al. (106)	Did not meet review criteria as participants had intermittent exotropia rather than myopia.
Yang et al. (107)	Did not meet review criteria as participants had intermittent exotropia rather than myopia.
Trbovich et al. (108)	Did not meet review criteria as participants had concussion-related receded near point of convergence.
Rovira-Gay et al. (109)	Did not meet review criteria as participants were adults with typical binocular vision rather than myopic children or adolescents.
Fazzi et al. (110)	Did not meet review criteria as participants were infants with visual impairment rather than school-aged children with myopia.
Abernethy and Wood (111)	Did not meet review criteria as this was a sports vision training study without myopia-related outcomes.
Sun et al. (43)	Full text unavailable.
Zhu et al. (112)	Full text unavailable.
Liu et al. (19)	Full text unavailable.
Olawade et al. (138)	Full text unavailable.
Sah et al. (113)	Full text unavailable.
Vivekanand et al. (114)	Full text unavailable.
Sreenivasan et al. (115)	Full text unavailable.
Chen et al. (116)	Intervention was light exposure rather than accommodative or visual training.
Chen et al. (117)	Intervention was traditional Chinese medicine therapy rather than accommodative or visual training.
Hu and Lin (118)	Insufficient controlled intervention data.
Jiang (119)	Not a controlled intervention study.
Li (120)	No comparable control group.
Li (49)	Master’s thesis, not a peer-reviewed journal article.
Liang et al. (121)	No comparable control group and outcomes were not focused on myopia control.
Liu et al. (122)	Combined multiple interventions and the effect of accommodative training could not be isolated.
Liu et al. (124)	Intervention was not accommodative or visual training.
Mi et al. (125)	Included pharmacological treatment and did not match the target intervention.
Yue et al. (126)	Intervention did not match the predefined visual training criteria.
Yue et al. (127)	Participants had pseudomyopia rather than myopia.
Chang et al. (128)	Outcomes were not related to myopia.
Horie et al. (129)	No eligible control group.
Yin et al. (22)	Duplicate or related report of an included study.
Jiang (130)	Correlation study; not an intervention.
Le et al. (131)	Observational study; not an intervention.
Luo et al. (132)	Observational study; not an intervention.
Xu et al. (133)	Case–control study; not an intervention.
Wang and Zhao (134)	Observational study; not an intervention.
Wang et al. (135)	Observational study; not an intervention.
Yang (136)	Clinical report; not an intervention.

Overall, the risk of bias in the included studies was mainly concentrated in the randomization process, reporting of results, measurement of outcomes, and control of confounding in non-randomized research. Therefore, the intervention effects in this review were interpreted cautiously in relation to the risk-of-bias level of each study.

### 3.3 Participant characteristics

The overall participant characteristics of the 13 studies included in this review were assessed as follows: (1) Sample size: the 13 studies involved approximately 1,413 participants in total, with ages mainly ranging from 6 to 18 years. The average sample size was approximately 109 participants per study, and the median sample size was 93 participants. (2) Sex: most of the included studies enrolled both male and female participants. Among the 13 studies, nine explicitly reported the sex distribution of participants, one reported only the proportion of female participants in each group without providing the exact numbers of male and female participants, and the remaining three studies did not clearly report sex distribution. (3) Age: the study populations mainly consisted of children and adolescents, with ages concentrated between 6 and 18 years. The median age was 10.5 years, and the mean age was 10.6 years.

### 3.4 Intervention characteristics

The interventions in the studies included in this review consisted of accommodative visual training and accommodation-related visual interventions embedded in physical activity. Accommodative visual training was the most common intervention type, mainly including VR/AR-based visual training (28, 33), computerized naked-eye three-dimensional visual training (27), eye exercises, and device-assisted ciliary muscle training (15, 34–36). Exercise-related interventions mainly involved the integration of visual recognition, near–far fixation switching, or kinetic visual acuity tasks into physical education classes, ball-sport courses, or specialized sports activities (20–25, 34). The intervention settings included home-based training, school physical education classes, extracurricular specialized sports activities, and device-assisted training environments. The duration of the trials ranged from immediate interventions to 2 years, with most lasting between 10 weeks and 6 months. The most common training frequency was daily training or 2–3 sessions per week, and the duration of each session was generally 20–40 min.

All 13 included studies adopted a pre–post assessment design. Among them, 11 studies included a control group (CG) (20–24, 27, 28, 33–36). Of these studies, eight used a single experimental group (EG) and control group (CG) design (20, 24, 27, 28, 33–36), whereas three included multiple experimental groups along with a control group (21–23). In addition, one study compared only two exercise intervention groups (25), and one study used a factorial clinical trial design (15).

### 3.5 Outcomes and measurements

#### 3.5.1 Effects of near–far accommodation-based exercise and visual training on ocular structural indicators and refractive progression

Among the included studies, nine reported indicators related to ocular structure or refractive progression. Ocular structural indicators mainly included axial length (AL) (21, 23, 27, 28, 34), spherical equivalent refraction (SE/SER) (21, 27), macular choroidal thickness or choroidal thickness (mCT/ChT) (28, 33), corneal curvature, and the axial length-to-corneal radius ratio (AL/CR) (21). Among these outcomes, AL and spherical equivalent refraction are the most clinically meaningful outcomes for myopia control.

Five studies reported findings suggesting that near–far accommodative visual training may help slow axial elongation. Xu et al. (28) applied virtual reality-based visual training (VRVT) combined with single-vision spectacles in children with low myopia. The results showed that, after 3 months of intervention, AL increased by 0.063 ± 0.060 mm in the VRVT group and by 0.129 ± 0.060 mm in the control group. The change-score MD was −0.033 mm (95% CI: −0.064 to −0.002, *p* = 0.037), indicating that AL progression was lower in the VRVT group than in the single-vision spectacle control group. However, at the 6-month follow-up, the between-group difference in AL was no longer significant (*p* = 0.172). Xie et al. (27) used computerized naked-eye three-dimensional visual training. After 6 months of intervention, AL increased by 0.176 mm in the training group and by 0.232 mm in the control group. The change-score MD was −0.056 mm (95% CI: −0.085 to −0.027, *p* < 0.001), indicating that AL progression was lower in the training group than in the control group. Zhou et al. (23) embedded ciliary muscle training with different visual target presentation durations into physical education classes. The results showed that AL increased in all groups after 32 weeks; however, the percentage increase in AL was lower in the 1-s and 3-s groups, with a between-group MD in change of −0.04 mm (*p* < 0.05). Xie et al. (34) added physiological eye-exercise training on the basis of near-use convex-lens prism treatment. After 2 years, AL increased by 0.23 ± 0.21 mm in the observation group and by 0.32 ± 0.19 mm in the control group. The change-score MD was −0.09 mm (95% CI: −0.166 to −0.014, *p* = 0.023), indicating that axial elongation was lower in the observation group than in the control group. In the study by Lin et al. (21), different small-ball sport interventions showed between-group differences in some AL-related indicators (*p* < 0.05). Comparisons among the table tennis, badminton, and control groups showed that AL increased in all three groups after the intervention; however, left-eye AL and AL/CR in the table tennis group were lower than those in the control group (*p* < 0.05), with endpoint MDs of −0.51 mm (95% CI, −0.986 to −0.034) and −0.58 mm (95% CI, −0.974 to −0.186), respectively.

Regarding refractive progression, Xie et al. (27) reported changes in SER. The change in SER was −0.251 D in the naked-eye three-dimensional visual training group and −0.354 D in the control group, with a between-group mean difference in change (change-score MD) of 0.103 D (95% CI: 0.019 to 0.188, *p* = 0.02). These findings indicated that the naked-eye three-dimensional visual training group showed a smaller negative shift in SER than the control group. Xie et al. (34) reported that refractive error changed by −0.81 ± 0.42 D in the observation group and by −1.17 ± 0.46 D in the control group. The between-group mean difference in change (change-score MD) was 0.36 D (95% CI: 0.192 to 0.528, *p* = 0.0001), suggesting that the combined training group showed less negative refractive progression. In the study by Lin et al. (21), after 6 months of small-ball sport interventions, the endpoint values of left-eye AL in the table tennis and badminton groups were lower than those in the control group, with endpoint MDs of −0.51 mm (95% CI: −0.986 to −0.034) and −0.58 mm (95% CI: −0.974 to −0.186), respectively. Binocular SE in the table tennis group was higher than that in the control group (*p* < 0.05), and left-eye SE in the badminton group was higher than that in the control group (*p* < 0.05). Wang et al. (24) reported that, after orienteering practice, refractive error in the experimental group changed from −3.35 ± 0.92 D to −2.99 ± 0.25 D (*Δ* + 0.36 D, *p* < 0.01).

For other indicators, Xu et al. (28) showed that mCT increased after 3 months of VR-based visual training (p < 0.05). The change in mCT was 22.633 ± 36.171 μm in the VRVT group and −0.301 ± 31.056 μm in the control group, with a between-group mean difference in change (change-score MD) of 25.633 μm (95% CI: 8.202 to 43.065, *p* = 0.005). However, at the 6-month follow-up, the between-group difference in mCT was no longer significant. Hu et al. (33) found a short-term decrease in ChT in the 0–1 mm zone after a single session of AR-based visual training (*p* = 0.007). The directions of change in choroidal indicators were inconsistent between the two studies, and the study by Hu et al. (33) examined only immediate effects; therefore, it cannot be directly interpreted as evidence for long-term myopia control. Accordingly, the current findings related to choroidal thickness should be regarded only as supplementary structural observations, and no stable or consistent conclusion regarding improvement can yet be drawn.

#### 3.5.2 Effects of near–far accommodation-based exercise and visual training on visual performance indicators

Visual performance indicators refer to measures that directly reflect an individual’s functional performance in visual tasks. These indicators mainly include uncorrected visual acuity, static visual acuity, dynamic visual acuity, kinetic visual acuity, contrast sensitivity, stereopsis, peripheral vision, visual reaction time, and eye–hand coordination. In the field of sports vision, static visual acuity, dynamic visual acuity, peripheral vision, spatial localization, eye–limb coordination, and visual reaction speed are also considered important components of visual performance capacity in sports contexts (37).

Among the included studies, nine reported indicators related to visual performance. These mainly included uncorrected distance visual acuity (UDVA) (22–25, 28, 35, 36), kinetic visual acuity (KVA) (22, 23, 25), mean visual acuity of the left and right eyes (20, 36), and the detection rate of poor vision (20). Overall, after near–far accommodative training and related visual–motor interventions, indicators such as uncorrected visual acuity and kinetic visual acuity generally showed a trend toward improvement.

In visual training studies, Xu et al. (28) applied VR-based visual training combined with single-vision spectacles in children with low myopia. After 3 months of intervention, the between-group mean difference in change (change-score MD) for UCVA (LogMAR) was −0.163 (95% CI: −0.236 to −0.090, *p* < 0.001). At the 6-month follow-up, the change-score MD for UCVA remained −0.133 (95% CI: −0.215 to −0.052, *p* = 0.002), indicating a greater improvement in uncorrected visual acuity in the VRVT group. This study also reported improvements in NVA. The between-group change-score MD for NVA was −0.053 at 3 months (95% CI: −0.103 to −0.004, *p* = 0.034) and −0.063 at 6 months (95% CI: −0.111 to −0.016, *p* = 0.010).

In studies of physical activity-embedded near–far accommodative training, Yin et al. (22) incorporated a physical activity program designed according to the principles of ciliary muscle training into regular physical activity and set different training frequencies of 15, 30, and 60 sessions. The results showed that KVA and UDVA improved significantly after the intervention in the 30-session and 60-session groups (*p* < 0.05), with the greatest improvement observed in the 30-session group. No significant changes in KVA or UDVA were observed in the 15-session group, whereas both indicators declined significantly in the control group (*p* < 0.05). Zhou et al. (23) embedded ciliary muscle training with different visual target presentation durations into physical education classes. After 32 weeks of intervention, KVA, UDVA, and AF improved in all experimental groups (*p* < 0.05). AL increased in all groups, but the percentage increase was lower in the 1-s and 3-s groups (*p* < 0.05). Zhao et al. (35) used the SU-001 ciliary muscle training device to intervene in early myopia among primary school students. The results showed that uncorrected visual acuity improved in the treatment group, whereas no obvious change was observed in the control group. Jin et al. (25) compared two physical activity interventions, cheerleading and football. The results showed that both groups improved in KVA and uncorrected visual acuity (*p* < 0.05). The improvement in KVA was greater in the football group than in the cheerleading group (*p* < 0.05), whereas the improvement in uncorrected visual acuity was greater in the cheerleading group than in the football group (*p* < 0.05).

In addition to the above studies, three studies of comprehensive exercise or exercise-prescription interventions also reported improvements in indicators such as left-eye and right-eye visual acuity, binocular visual acuity, mean visual acuity, and the detection rate of poor vision. Zheng et al. (36) reported that, after 6 months of intervention, the endpoint values of left-eye, right-eye, and binocular uncorrected visual acuity were higher in the experimental group than in the control group. The endpoint MD was 0.380 for the left eye (95% CI: 0.358 to 0.402, *p* < 0.01), 0.364 for the right eye (95% CI: 0.339 to 0.389, *p* < 0.01), and 0.415 for binocular visual acuity (95% CI: 0.391 to 0.439, *p* < 0.01), indicating improvements in left-eye, right-eye, and binocular visual acuity in the experimental group. In a tennis intervention experiment, Han et al. (20) reported that the mean visual acuity of the left and right eyes was higher in the intervention group than in the control group (*p* < 0.01), and the detection rate of poor vision was lower than that in the control group (*p* < 0.01). Wang et al. (24) reported that, after 10 weeks of orienteering practice, uncorrected visual acuity and refractive error in the experimental group improved compared with baseline, and the original article reported statistically significant differences (*p* < 0.01).

#### 3.5.3 Effects of near–far accommodation-based exercise and visual training on accommodative function indicators

A total of five studies examined the effects of near–far accommodative training or accommodation-related visual training on accommodative function indicators. These indicators included accommodative facility (AF) (15, 21, 23, 33), amplitude of accommodation (AMP) (21), accommodative response (15), accommodative lag (15), and negative relative accommodation (NRA) and positive relative accommodation (PRA) (21).

Regarding accommodative facility (AF), Zhou et al. (23) embedded ciliary muscle training with different visual target presentation durations into physical education classes. After 32 weeks of intervention, AF improved significantly in all experimental groups (*p* < 0.05), accompanied by improvements in KVA (*p* < 0.05) and UDVA (*p* < 0.05). Allen et al. (15) used a visual training program combined with contact lenses designed to manipulate spherical aberration. The results showed that distance accommodative facility improved significantly after visual training (*p* < 0.05), whereas near accommodative facility improved only in the training group (*p* < 0.05). Hu et al. (33) reported that, after a single session of AR-based visual training, monocular AF in the AR group increased from 5.05 ± 1.38 cpm to 8.05 ± 1.81 cpm (*Δ* +3.00 cpm, 95% CI: 1.83 to 4.17, *p* = 0.004), whereas the change in binocular AF was not statistically significant (*p* = 0.127). Lin et al. (21) provided evidence from the perspective of small-ball sport interventions. The results showed that, after 6 months of moderate-intensity table tennis exercise, the endpoint between-group mean difference in binocular AF for the table tennis group was 0.59 cpm (95% CI: 0.102 to 1.078), indicating an improvement compared with baseline (*p* < 0.05). Binocular AF also improved in the badminton group and was higher than that in the control group.

Regarding amplitude of accommodation and relative accommodation indicators, Lin et al. (21) reported that the badminton group showed an improvement in right-eye amplitude of accommodation and binocular accommodative facility (*p* < 0.05), whereas positive relative accommodation (PRA) decreased (*p* < 0.05). Although negative relative accommodation (NRA) was included as an outcome measure, the currently extracted results did not show a clear improvement. Allen et al. (15) reported that spherical-aberration-modified contact lenses reduced near accommodative lag (*p* < 0.05). After visual training, distance positive and negative accommodative response times were shortened (*p* < 0.05), whereas near negative response time was shortened only in the training group (*p* < 0.05).

## 4 Discussion

This study aimed to systematically evaluate the effects of near–far accommodative training and related visual–motor interventions on visual function and myopia-related indicators in children and adolescents, and to explore the potential role of these interventions in myopia prevention and control. Overall, the findings from the 13 included studies suggest that these interventions showed relatively consistent trends toward improvement in indicators such as uncorrected visual acuity, kinetic visual acuity, and accommodative facility. Some studies also suggested that these interventions may help slow axial elongation and reduce the negative shift in spherical equivalent refraction. It should be emphasized that different outcome indicators carry different levels of clinical relevance and interpretive weight. Axial length and spherical equivalent refraction reflect structural and refractive changes during the onset and progression of myopia and were therefore regarded in this review as the core clinical outcomes for evaluating myopia control effects. By contrast, uncorrected visual acuity, kinetic visual acuity, and accommodative function indicators were considered supportive evidence for improvements in visual function and for exploring potential mechanisms of action.

### 4.1 Effects of near–far accommodation-based exercise and visual training on ocular structural indicators and refractive progression

Axial length reflects developmental changes in ocular structure, whereas spherical equivalent refraction can accurately characterize refractive status. Indicators of ocular structure and refractive progression are objective outcomes for evaluating whether near–far accommodative training has relevance for myopia prevention and control, as they more directly reflect the biological changes underlying the onset and progression of myopia. The structural basis of myopia progression is primarily manifested as excessive axial elongation. The International Myopia Institute (2) has also noted that axial elongation is an important factor driving myopia progression, and that refractive error and axial length are commonly used as primary outcome measures in clinical trials of myopia control.

The development of myopia is often accompanied by impairment or dysregulation of ciliary muscle accommodative function (23). Studies have shown that specific accommodative exercises can enhance contraction and relaxation of the ciliary muscle, thereby improving ocular perfusion and modulating retinal defocus stimuli (38). The effects of near–far accommodative training on axial elongation and refractive progression may be mediated mainly through improvements in accommodative function and reductions in retinal defocus stimuli. From the perspective of retinal defocus theory, when the accommodative response is insufficient during near fixation, the image focus falls behind the retina, producing hyperopic retinal defocus. This may generate visual signals that promote axial elongation (11, 13, 39). This perspective provides a rationale for studies emphasizing accommodative training (15, 27, 28, 33–36) and sports training with embedded accommodative components (20–25) in the prevention of myopia among children and adolescents.

In the study by Xu et al. (28), VRVT simulated changes in near and far viewing distances and incorporated ciliary muscle accommodative activities. This training may enhance the eyes’ ability to focus on targets at different distances and thereby reduce hyperopic retinal defocus stimuli during near tasks. In addition, increased choroidal thickness may produce a short-term anterior shift of the retinal position, helping to reduce defocus error signals; this may partly explain the short-term slowing of axial elongation observed in that study. The study by Xie et al. (34) also supports this interpretation. Their intervention combined near-use convex-lens prisms with physiological visual eye exercises. The near-use convex-lens prism may reduce accommodative demand during near work to some extent and improve binocular convergence and fusion status. When combined with physiological visual eye exercises, this intervention may promote regular alternation between accommodative tension and relaxation. Such training may help relieve sustained ciliary muscle contraction, reduce residual accommodation and near-work-induced transient myopia, and thereby lower the risk of further negative refractive progression. In sports contexts, near–far accommodative training can be implemented through various ball games. Table tennis and badminton require children and adolescents to continuously track balls moving in different directions, at different speeds, and across varying distances. These visual demands may help relieve accommodative tension and improve amplitude of accommodation, accommodative facility, and accommodative reserve. Improved accommodative function may reduce accommodative lag and hyperopic defocus stimuli, thereby helping to slow axial elongation and myopia progression to some extent (21). Small-ball sports such as table tennis and badminton involve fast ball speeds and frequent changes in direction. Participants must continuously track and judge the trajectory of the ball, which repeatedly stimulates refractive accommodation and ciliary muscle contraction and relaxation. This may be more favorable for myopia prevention and control, consistent with the findings reported by Hu (40).

### 4.2 Effects of near–far accommodation-based exercise and visual training on visual performance indicators

The functional visual impact caused by myopia can be reflected by visual performance indicators such as uncorrected visual acuity and kinetic visual acuity (41). Static uncorrected visual acuity and kinetic visual acuity reflect an individual’s ability to identify target details under static or dynamic visual conditions. In the present review, most studies (*n* = 9) focused on indicators such as uncorrected visual acuity or kinetic visual acuity, which is generally consistent with the outcome selection used in previous related studies (41, 42).

Near–far accommodative training showed a significant trend toward improvement in uncorrected visual acuity and kinetic visual acuity. KVA, defined as the ability to perceive details of objects moving toward or away from the eyes, is highly dependent on the ciliary muscle and closely related to accommodative function (22, 23). Kinetic visual acuity training mainly involves visual–motor target tasks such as forward–backward movement, changes in speed, and continuous multi-ball tasks. These tasks require participants to rapidly accommodate, focus, judge, and refocus as the target continuously changes (22, 23, 25). Dynamic visual acuity is positively correlated with static visual acuity; therefore, strengthening dynamic visual acuity training may contribute to improvements in static visual acuity (43).

Training centered on ciliary muscle contraction and relaxation may help relieve accommodative tension after sustained near work and improve the clarity of distance retinal imaging (35). Emerging intelligent training approaches, such as VR-based visual training, can also construct tasks that simulate near–far visual activities through virtual reality scenarios (28). Such training may not only improve short-term visual clarity but may also further influence ocular biological indicators related to myopia progression through improvements in accommodative function. Studies of naked-eye three-dimensional vision training have also shown that, after 6 months of training, axial elongation and the negative progression of SER were lower in the intervention group than in the control group (27). These findings suggest that ciliary muscle training devices, comprehensive VR-based visual training, and naked-eye three-dimensional vision training share a common feature: they repeatedly generate visual stimuli with different refractive demands, thereby strengthening accommodative function and improving the clarity and stability of retinal imaging. Visual training in sports contexts further demonstrates the intervention effects of near–far accommodative training on visual performance (20, 22–25, 36, 137). These findings are consistent with previous meta-analytic evidence (38), which suggests that near–far accommodative training and visual accommodative interventions embedded in physical activity have positive effects on visual performance indicators in children and adolescents. Embedding ciliary muscle training into physical activity or PE classes may improve children’s kinetic visual acuity, uncorrected distance visual acuity, and accommodative facility. In other words, when physical activity includes near–far target recognition, visual refocusing, and dynamic tracking tasks, it may enhance the visual system’s ability to clearly identify targets at different distances (22, 23).

### 4.3 Effects of near–far accommodation-based exercise and visual training on accommodative function indicators

Accommodative facility refers to the ability of the eye to rapidly shift between different refractive stimuli and regain clear retinal imaging. Clinically, it is commonly assessed using ±2.00 D plus/minus lenses by recording the number of accommodative cycles completed within a given period, thereby reflecting the dynamic switching efficiency of the accommodative system (44). A greater number of cycles completed within 1 min indicates better accommodative function.

There is consistency in the underlying functional pathway between accommodative facility testing and near–far accommodative training. AF testing repeatedly alternates between plus and minus refractive stimuli, requiring participants to continuously increase accommodation, relax accommodation, and regain clear vision. Therefore, the “facility” component primarily reflects the speed of switching between plus and minus lenses and the time required to regain clarity, namely positive and negative accommodative response times, rather than simply the magnitude of accommodation (45, 46). Individuals with higher AF may identify defocus signals more rapidly. The supraoculomotor area (SOA) in the midbrain can then more promptly regulate the activity of preganglionic parasympathetic neurons in the Edinger–Westphal nucleus. Through the oculomotor nerve, ciliary ganglion, and short ciliary nerves, this pathway rapidly modulates ciliary muscle tone, enabling the crystalline lens to increase or decrease its refractive power more efficiently (47, 48). The effect of near–far accommodative training on accommodative facility (AF) lies in its repeated reinforcement of the accommodative system’s dynamic switching ability across different refractive demands. Embedding ciliary muscle training into physical activity or PE classes, with different training frequencies and different visual target presentation durations, allows children to repeatedly perform visual target recognition and near–far accommodative shifts during movement, which may effectively improve AF (22, 23). Similarly, AR-based visual training can stimulate AF through changes in target depth and size (33). These training approaches may enhance accommodative facility by repeatedly creating rapid and accurate near–far visual switching tasks in sports or digital visual environments. Other related studies have also discussed the role of near–far accommodative training and visual accommodative interventions embedded in physical activity in myopia prevention and control (49). Such interventions may strengthen accommodative ability and potentially exert positive effects on myopia prevention and control.

According to the principle of training specificity, when the training stimulus is highly consistent with the testing task in terms of movement structure and functional demands, the sensitivity of the trained accommodative response is more likely to transfer to test performance (50). Therefore, after near–far accommodative training, AF may show a relatively large improvement during testing.

## 5 Limitations

This review has several limitations. First, the reporting of outcome indicators was not balanced across the included studies. In particular, indicators such as accommodative lag, amplitude of accommodation, and positive and negative relative accommodation were reported in only a small number of studies. This may have weakened the ability of this review to provide a comprehensive explanation of the mechanisms underlying near–far accommodative training and also limited further interpretation of the internal relationships among different outcome indicators. Second, some studies did not provide detailed information on participants’ sex distribution, baseline degree of myopia, refractive status, or history of previous interventions. This may have affected the analysis of differences in responses to near–far accommodative training across different populations. Finally, this review included only studies published in Chinese and English, and relevant studies published in other languages may therefore have been missed.

## 6 Conclusion

The findings of this review suggest that near–far accommodation-based exercise interventions and visual training may be associated with improvements in certain myopia-related indicators in children and adolescents. Across the existing studies, relatively consistent improvements were observed in visual function and accommodative function indicators, such as uncorrected visual acuity, dynamic visual acuity, and accommodative facility. These findings suggest that such interventions may help enhance near–far focusing shifts, dynamic target tracking, and accommodative response capacity. However, the interpretation of their effects on myopia prevention and control should remain cautious. Axial length and cycloplegic spherical equivalent refraction are the core outcomes with greater clinical relevance for evaluating myopia progression. Although some studies reported slower axial elongation or less negative progression of spherical equivalent refraction after intervention, the number of studies reporting these core outcomes remains limited. In addition, the included studies differed in intervention protocols, training dose, measurement methods, follow-up duration, and methodological quality. Therefore, the current evidence should be interpreted as preliminary and suggestive rather than confirmatory evidence that these interventions can consistently control myopia progression in children and adolescents. For supplementary indicators such as choroidal thickness, corneal curvature, AL/CR ratio, and visual fatigue, the current findings remain inconsistent and should not yet be used as the primary basis for judging long-term myopia-control effects. Future studies should conduct well-designed randomized controlled trials with adequate follow-up, adopt standardized intervention protocols, and report core outcomes such as axial length and cycloplegic spherical equivalent refraction, while also examining the relationships among visual performance, accommodative function, and refractive progression.

## Data Availability

The original contributions presented in the study are included in the article/Supplementary material, further inquiries can be directed to the corresponding author.

## References

[1] Flitcroft DI He M Jonas JB Jong M Naidoo K Ohno-Matsui K . IMI—defining and classifying myopia: a proposed set of standards for clinical and epidemiologic studies. Invest Ophthalmol Vis Sci. (2019) 60:M30. doi: 10.1167/iovs.18-25957 PMC6735818 30817826

[2] International Myopia Institute (2019). Myopia is growing around the world: what is myopia? Available online at: https://myopiainstitute.org/myopia/ (accessed June 21, 2026).

[3] Subudhi P. Agarwal P. (2023). Myopia. In: StatPearls. Treasure Island: StatPearls Publishing. Available online at: https://www.ncbi.nlm.nih.gov/books/NBK580529/?report=printable (Accessed June 21, 2026).

[4] Holden BA Fricke TR Wilson DA Jong M Naidoo KS Sankaridurg P . Global prevalence of myopia and high myopia and temporal trends from 2000 through 2050. Ophthalmology. (2016) 123:1036–42. doi: 10.1016/j.ophtha.2016.01.006 26875007

[5] Liang JH Pu YQ Chen JQ Liu M Ouyang B Jin Z . Global prevalence, trend and projection of myopia in children and adolescents from 1990 to 2050: a comprehensive systematic review and meta-analysis. Br J Ophthalmol. (2025) 109:362–71. doi: 10.1136/bjo-2024-325427 39317432

[6] Bullimore MA Brennan NA. Myopia control: why each diopter matters. Optom Vis Sci. (2019) 96:463–5. doi: 10.1097/OPX.0000000000001367 31116165

[7] Haarman AEG Enthoven CA Tideman JWL Tedja MS Verhoeven VJM Klaver CCW. The complications of myopia: a review and meta-analysis. Invest Ophthalmol Vis Sci. (2020) 61:49. doi: 10.1167/iovs.61.4.49 PMC7401976 32347918

[8] He M Xiang F Zeng Y Mai J Chen Q Zhang J . Effect of time spent outdoors at school on the development of myopia among children in China: a randomized clinical trial. JAMA. (2015) 314:1142–8. doi: 10.1001/jama.2015.10803 26372583

[9] Morgan IG Wu PC Ostrin LA Tideman JWL Yam JC Lan W . IMI risk factors for myopia. Invest Ophthalmol Vis Sci. (2021) 62:3. doi: 10.1167/iovs.62.5.3 PMC8083079 33909035

[10] Flitcroft DI. The complex interactions of retinal, optical and environmental factors in myopia aetiology. Prog Retin Eye Res. (2012) 31:622–60. doi: 10.1016/j.preteyeres.2012.06.004 22772022

[11] Gwiazda J Bauer J Thorn F Held R. A dynamic relationship between myopia and blur-driven accommodation in school-aged children. Vis Res. (1995) 35:1299–304. doi: 10.1016/0042-6989(94)00238-H 7610590

[12] Kaphle D Varnas SR Schmid KL Suheimat M Leube A Atchison DA. Accommodation lags are higher in myopia than in emmetropia: measurement methods and metrics matter. Ophthalmic Physiol Opt. (2022) 42:1103–14. doi: 10.1111/opo.13021 35775299 PMC9544228

[13] Logan NS Radhakrishnan H Cruickshank FE Allen PM Bandela PK Davies LN . IMI accommodation and binocular vision in myopia development and progression. Invest Ophthalmol Vis Sci. (2021) 62:4. doi: 10.1167/iovs.62.5.4 PMC8083074 33909034

[14] Prousali E Haidich AB Tzamalis A Ziakas N Mataftsi A. The role of accommodative function in myopic development: a review. Semin Ophthalmol. (2022) 37:455–61. doi: 10.1080/08820538.2021.2006724 34821535

[15] Allen PM Radhakrishnan H Rae S Calver RI Theagarayan B Price H . Aberration control and vision training as an effective means of improving accommodation in individuals with myopia. Invest Ophthalmol Vis Sci. (2009) 50:5120–9. doi: 10.1167/iovs.08-2865 19643961

[16] Huang Y Li M Shen Y Liu F Fang Y Xu H . Study of the immediate effects of autostereoscopic 3D visual training on the accommodative functions of myopes. Invest Ophthalmol Vis Sci. (2022) 63:9. doi: 10.1167/iovs.63.2.9 PMC8819359 35113140

[17] Ma MML Shi JC Li N Scheiman M Chen X. Effect of vision therapy on accommodative lag in myopic children: a randomized clinical trial. Optom Vis Sci. (2019) 96:17–26. doi: 10.1097/OPX.0000000000001316 30575616

[18] Lochhead L Feng J Laby DM Appelbaum LG. Visual performance and sports: a scoping review. J Sport Exerc Psychol. (2024) 46:205–17. doi: 10.1123/jsep.2023-0267 38815973

[19] Liu JM Lan WC Zhang DX. Network meta-analysis of the efficacy of physical exercise interventions on vision health in children and adolescents. Front Public Health. (2024) 12:1393909. doi: 10.3389/fpubh.2024.1393909 39267655 PMC11390692

[20] Han JM Fan ZM Wang HH Qiu C Rao ZL Liu Y . Empirical study on the effect of tennis exercise on visual health in primary school students (in Chinese). Chin J Sch Health. (2023) 44:1804–8. doi: 10.16835/j.cnki.1000-9817.2023.12.010

[21] Lin WT Li YX Peng YQ Huang Y Diao YH Chen X . Experimental study on the effects of table tennis and badminton exercise on myopia prevention and control in children aged 7–9 years (in Chinese). Chin J Sports Med. (2025) 44:13–21. doi: 10.16038/j.1000-6710.2025.01.006

[22] Yin R Xu J Wang H Zhou S Zhang M Cai G. Effect of physical activity combined with extra ciliary-muscle training on visual acuity of children aged 10–11. Front Public Health. (2022) 10:949130. doi: 10.3389/fpubh.2022.949130 36111187 PMC9468474

[23] Zhou S Zhang M Zheng W Yin R Chen G. Effects of physical activity combined with different visual target presentation durations of ciliary-muscle training on visual acuity in children. Front Public Health. (2023) 11:1191112. doi: 10.3389/fpubh.2023.1191112 37538276 PMC10394291

[24] Wang L Liu JR Liu Y. Evaluation of the intervention effect of orienteering exercise on myopia prevention and control in primary school students (in Chinese). Chin J Sch Health. (2023) 44:521–4. doi: 10.16835/j.cnki.1000-9817.2023.04.010

[25] Jin G Pan JL Cai G. Practical significance and empirical research on physical exercise for visual health in primary school students (in Chinese). J Capital Univ Phys Educ Sports. (2021) 33:40–8. doi: 10.14036/j.cnki.cn11-4513.2021.01.005

[26] Brennan NA Toubouti YM Cheng X Bullimore MA. Efficacy in myopia control. Prog Retin Eye Res. (2021) 83:100923. doi: 10.1016/j.preteyeres.2020.100923 33253901

[27] Xie R Zhao F Yu J Luo B Jiang Z Qiu X . Naked-eye 3-dimensional vision training for myopia control: a randomized clinical trial. JAMA Pediatr. (2024) 178:533–9. doi: 10.1001/jamapediatrics.2024.0578 38587852 PMC11148688

[28] Xu Z Zou A Li L Wu Y Cai W Ma J . Effect of virtual reality-based visual training for myopia control in children: a randomized controlled trial. BMC Ophthalmol. (2024) 24:358. doi: 10.1186/s12886-024-03580-w 39278928 PMC11404007

[29] Moher D Liberati A Tetzlaff J Altman DG PRISMA Group. Preferred reporting items for systematic reviews and meta-analyses: the PRISMA statement. PLoS Med. (2009) 6:e1000097. doi: 10.1371/journal.pmed.1000097 21603045 PMC3090117

[30] Page MJ McKenzie JE Bossuyt PM Boutron I Hoffmann TC Mulrow CD . The PRISMA 2020 statement: an updated guideline for reporting systematic reviews. PLoS Med. (2021) 18:e1003580. doi: 10.1371/journal.pmed.1003580 33780438 PMC8007028

[31] Sterne JAC Savović J Page MJ Elbers RG Blencowe NS Boutron I . RoB 2: a revised tool for assessing risk of bias in randomised trials. BMJ. (2019) 366:l4898. doi: 10.1136/bmj.l4898 31462531

[32] Sterne JAC Hernán MA Reeves BC Savović J Berkman ND Viswanathan M . ROBINS-I: a tool for assessing risk of bias in non-randomised studies of interventions. BMJ. (2016) 355:i4919. doi: 10.1136/bmj.i4919 27733354 PMC5062054

[33] Hu GR Xu ZX Pan CF Tao ZY Chu H Lv ZY . Immediate impact of augmented reality visual training on choroidal thickness and accommodative function: a small sample study. Photodiagn Photodyn Ther. (2025) 55:104756. doi: 10.1016/j.pdpdt.2025.104756 40782879

[34] Xie XY He BH Du HJ Cheng Z. Clinical significance of convex-lens prism combined with physiological visual eye exercises for the prevention and control of adolescent myopia (in Chinese). Recent Adv Ophthalmol. (2013) 33:475–7. doi: 10.13389/j.cnki.rao.2013.05.022

[35] Zhao WR Zhao HH Ma WH Huo JF Zhang XM Cao X . Therapeutic effects of ciliary muscle training on early myopia in primary school students (in Chinese). Chin J Rehabil Med. (2008) 23:153–4. doi: 10.3969/j.issn.1001-1242.2008.02.018

[36] Zheng BJ Li YH Liao XH Zhang Y Zhang D Zhong L . Intervention effects of physical exercise integrated with traditional Chinese medicine regulation on myopia in children and adolescents (in Chinese). J Capital Univ Phys Educ Sports. (2022) 34:538–44. doi: 10.14036/j.cnki.cn11-4513.2022.05.009

[37] Buscemi A Mondelli F Biagini I Gueli S D'Agostino A Coco M. Role of sport vision in performance: systematic review. J Funct Morphol Kinesiol. (2024) 9:92. doi: 10.3390/jfmk9020092 38921628 PMC11204951

[38] Nie P Feng T. Meta-analysis of the effects of physical activity on ocular biometrics in children and adolescents. Front Public Health. (2025) 13:1615033. doi: 10.3389/fpubh.2025.1615033 40567953 PMC12187683

[39] Smith EL Hung LF Arumugam B. Visual regulation of refractive development: insights from animal studies. Eye. (2014) 28:180–8. doi: 10.1038/eye.2013.277 24336296 PMC3930279

[40] Hu CX. Comparative analysis of the impact of table tennis, basketball, and middle- and long-distance running on vision in children and adolescents (in Chinese). Contemp Sports Technol. (2015) 5:210–1. doi: 10.16655/j.cnki.2095-2813.2015.01.131

[41] Zhao E Wang X Zhang H Zhao E Wang J Yang Y . Ocular biometrics and uncorrected visual acuity for detecting myopia in Chinese school students. Sci Rep. (2022) 12:18644. doi: 10.1038/s41598-022-23409-0 36333404 PMC9636232

[42] Wang Y Guo Y Wei S Yuan Y Wu T Zhang Y . Binocular dynamic visual acuity in eyeglass-corrected myopic patients. J Vis Exp. (2022) 181:e63864. doi: 10.3791/63864 35435897

[43] Sun L Cai G Yin RB Pan J Wang G Chen G . Correlation between static visual acuity and dynamic visual acuity in children and its significance for physical activity (in Chinese). Chin J Rehabil Theory Pract. (2018) 24:1485–8. doi: 10.3969/j.issn.1006-9771.2018.12.025

[44] Hilora M. Tripathy K. (2025). Accommodative excess. In: StatPearls. Treasure Island: StatPearls Publishing. Available online at: https://ncbi.nlm.nih.gov/books/NBK592379/?report=printable (accessed June 21, 2026). 37276314

[45] Allen PM Charman WN Radhakrishnan H. Changes in dynamics of accommodation after accommodative facility training in myopes and emmetropes. Vis Res. (2010) 50:947–55. doi: 10.1016/j.visres.2010.03.007 20304003

[46] Radhakrishnan H Allen PM Charman WN. Dynamics of accommodative facility in myopes. Invest Ophthalmol Vis Sci. (2007) 48:4375–82. doi: 10.1167/iovs.07-0269 17724230

[47] May PJ Warren S Gamlin PDR Billig I. An anatomic characterization of the midbrain near response neurons in the macaque monkey. Invest Ophthalmol Vis Sci. (2018) 59:1486–502. doi: 10.1167/iovs.17-23737 29625471 PMC5861931

[48] Motlagh M. Geetha R. (2022). Physiology, accommodation. StatPearls. Treasure Island: StatPearls Publishing. Available online at: https://ncbi.nlm.nih.gov/books/NBK542189/?report=printable (accessed June 21, 2026). 31194346

[49] Li L Xu JF Lu YL Feng L. Research progress on outdoor activities and physical exercise for the prevention and control of myopia in children and adolescents (in Chinese). China Sport Sci Technol. (2019) 55:3–13. doi: 10.16470/j.csst.2019029

[50] Morrissey MC Harman EA Johnson MJ. Resistance training modes: specificity and effectiveness. Med Sci Sports Exerc. (1995) 27:648–60. doi: 10.1249/00005768-199505000-00006 7674868

[51] He X Sankaridurg P Wang J Chen J Naduvilath T He M . Time outdoors in reducing myopia: a school-based cluster randomized trial with objective monitoring of outdoor time and light intensity. Ophthalmology. (2022) 129:1245–54. doi: 10.1016/j.ophtha.2022.06.024, 35779695

[52] Li JY Liu FR Zhou XW Li CA Huang HH. Outdoor exposure and myopia prevention and control in school-aged children.(in Chinese). Chin J Sch Health. (2018) 39:1227–9. doi: 10.16835/j.cnki.1000-9817.2018.08.032

[53] Ying ZQ Li DL Zheng XY Zhang XF Pan CW. Risk factors for myopia among children and adolescents: an umbrella review of published meta-analyses and systematic reviews. Br J Ophthalmol. (2024) 108:167–74. doi: 10.1136/bjo-2022-322773, 36754586

[54] Yu M Hu Y Han M Song J Wu Z Xu Z . Global risk factor analysis of myopia onset in children: a systematic review and meta-analysis. PLoS One. (2023) 18:e0291470. doi: 10.1371/journal.pone.0291470, 37729320 PMC10511087

[55] Morgan IG French AN Ashby RS Guo X Ding X He M . The epidemics of myopia: aetiology and prevention. Prog Retin Eye Res. (2018) 62:134–49. doi: 10.1016/j.preteyeres.2017.09.004, 28951126

[56] Dhakal R Shah R Huntjens B Verkicharla PK Lawrenson JG. Time spent outdoors as an intervention for myopia prevention and control in children: an overview of systematic reviews. Ophthalmic Physiol Opt. (2022) 42:545–58. doi: 10.1111/opo.12945, 35072278 PMC9305934

[57] Xiong S Sankaridurg P Naduvilath T Zang J Zou H Zhu J . Time spent in outdoor activities in relation to myopia prevention and control: a meta-analysis and systematic review. Acta Ophthalmol. (2017) 95:551–66. doi: 10.1111/aos.13403, 28251836 PMC5599950

[58] Cheng MC Zhang RL. Optimal exercise modality and dose for improving myopia in children and adolescents (in Chinese). J Capital Univ Phys Educ Sports. (2025) 37:155–63. doi: 10.14036/j.cnki.cn11-4513.2025.02.006

[59] Xie XL Li LP Wang B Ma J Chen Q Zhao HP (2025). Application of a nomogram in the association between visual behavior and myopia among preschool children (in Chinese). Recent Adv Ophthalmol 45, 539–545, 553.

[60] Zhang RZ Xu JF Zhang B Li FY Guo TY Li L. Association of physical exercise with myopia prevention and body-shape development in children: a 1-year longitudinal intervention study (in Chinese). China Sport Sci. (2025) 45:73–82. doi: 10.16469/J.css.2025KX005

[61] Xu S Ruan Z Wang Y Jiang J Zhao F Tang X . Establishment of a myopia occurrence prediction model in children without myopia using cycloplegic refraction and prior axial length change. Ophthalmology. (2025) 132:1260–72. doi: 10.1016/j.ophtha.2025.06.010, 40516576

[62] Read SA Collins MJ Vincent SJ. Light exposure and eye growth in childhood. Invest Ophthalmol Vis Sci. (2015) 56:6779–87. doi: 10.1167/iovs.14-15978, 26567790

[63] Fan JX Song R Liao JH . Effects of a badminton exercise prescription on myopia in adolescents under the context of sports–medicine integration (in Chinese). J Qiannan Norm Univ Natl. (2023) 43:107–15.

[64] Wu R. J. *Effects of Taekwondo on myopia prevention and control in primary school students based on machine-learning theory* (master’s thesis). Soochow University, Suzhou (2024)(in Chinese)

[65] Zhang W. S. *Therapeutic effects of combined badminton exercise and visual hygiene education on pseudomyopia in junior high school students* (master’s thesis). Shanxi Normal University, Shanxi (2021)(in Chinese)

[66] Nie P. F. *Effects of modified physical games on visual acuity in junior high school students* (master’s thesis). Soochow University, Suzhou (2021)(in Chinese)

[67] Zhou C. *Effects of basic track-and-field exercises with additional dynamic visual tasks on visual acuity in fourth-grade pupils with myopia* (master’s thesis). Soochow University, Suzhou (2021)(in Chinese)

[68] Yan X. *Effects of traditional active folk games on visual acuity in second-grade primary school children* (master’s thesis). Soochow University, Suzhou (2021)(in Chinese)

[69] Chen X. Y. *Experimental effects of table tennis and shuttlecock exercise on myopia in children and adolescents* (master’s thesis). Guangzhou Sport University, Guangzhou (2024)(in Chinese)

[70] Xue A Zeng Z Wang H Han J Pang B. Kinematic characteristics of gait with different myopia: a cross-sectional study. Front Public Health. (2023) 11:1256242. doi: 10.3389/fpubh.2023.1256242, 38179553 PMC10765516

[71] Huang Y. L. *Effects of table-tennis forehand and backhand exercises on visual acuity in children* (master’s thesis). Guangzhou Sport University, Guangzhou (2021)(in Chinese)

[72] Xie M. *Effects of football basic exercises with enhanced dynamic visual tasks on visual acuity in fifth-grade primary school pupils with myopia* (master’s thesis). Soochow University, Suzhou (2021)(in Chinese)

[73] Song SX Wang FY Li YH. Comparative study of the effects of table-tennis exercise on visual acuity in children and adolescents. (in Chinese). China Sport Sci Technol. (2002) 38:19–20. doi: 10.16470/j.csst.2002.11.006

[74] Chen GW Feng JC Chen HM. Cross-sectional study of dynamic visual acuity and years of table-tennis training. (in Chinese). Sports Sci Res. (2018) 22:66–70.

[75] Jouira G Alexe CI Zinelabidine K Rebai H Mocanu GD Cojocaru AM . The impact of aerobic dance intervention on postural balance in children: a randomized controlled trial. Children. (2024) 11:573. doi: 10.3390/children11050573, 38790568 PMC11120053

[76] Tao FB Pan CW Wu XY He X Guo X. Expert recommendations on outdoor activities for the prevention and control of myopia in children and adolescents (in Chinese). Chin J Sch Health. (2019) 40:641–3.

[77] Chen D Wang J Chen J Lu M du Y Zhu Z . Smartwatch-monitored physical activity and myopia in children: a 2-year prospective cohort study. BMC Med. (2025) 23:294. doi: 10.1186/s12916-025-04136-5, 40399891 PMC12096555

[78] Lundberg K Vestergaard AH Jacobsen N Suhr Thykjær A Søgaard Hansen R Goldschmidt E . Choroidal thickness and myopia in relation to physical activity—the CHAMPS eye study. Acta Ophthalmol. (2018) 96:371–8. doi: 10.1111/aos.13640, 29338123

[79] Li S Pan Y Xu J Li X Spiegel DP Bao J . Effects of physical exercise on macular vessel density and choroidal thickness in children. Sci Rep. (2021) 11:2015. doi: 10.1038/s41598-021-81770-y, 33479470 PMC7820247

[80] Mei Z Zhang Y Jiang W Lam C Luo S Cai C . Efficacy of outdoor interventions for myopia in children and adolescents: a systematic review and meta-analysis of randomized controlled trials. Front Public Health. (2024) 12:1452567. doi: 10.3389/fpubh.2024.1452567, 39193200 PMC11347293

[81] Al-Mohtaseb Z Schachter S Lee BS Shen Lee B Garlich J Trattler W. The relationship between dry eye disease and digital screen use. Clin Ophthalmol. (2021) 15:3811–20. doi: 10.2147/OPTH.S321591, 34531649 PMC8439964

[82] Wang X Yuan M Hu LJ Wang MJ Zhang MF. Effects of different ocular rest methods on alleviating visual fatigue in medical students (in Chinese). Chin J Sch Health. (2016) 37:1314–7.

[83] Yang DL Guo CY Zhou YF Yu J Wang PF Lu Q . Association between physical activity and myopia in primary and secondary school students (in Chinese). J Bio-Educ. (2016) 4:17–21.

[84] O’Donoghue L Kapetanankis VV McClelland JF Logan NS Owen CG Saunders KJ . Risk factors for childhood myopia: findings from the NICER study. Invest Ophthalmol Vis Sci. (2015) 56:1524–30. doi: 10.1167/iovs.14-15549, 25655799

[85] Liao S Li X Bai N Wu D Yang W Wang F . An empirical study on the effect of outdoor illumination and exercise intervention on children’s vision. Front Public Health. (2023) 11:1270826. doi: 10.3389/fpubh.2023.1270826, 38155899 PMC10754518

[86] Ma F Yang J Yuan J Du B Li T Wu Q . The myopia prevalence and association with physical activity among primary school students aged 6–12 years: a cross-sectional study in Tianjin, China. Transl Vis Sci Technol. (2024) 13:4. doi: 10.1167/tvst.13.6.4, 38864819 PMC11174138

[87] Lu Y Wang J Chen J Yan Y Zeng H Zhang B . Impact of environmental factors on short-term eye strain relief during COVID-19 quarantine: a pilot study. Forests. (2022) 13:1966. doi: 10.3390/f13111966

[88] Biswas S El Kareh A Qureshi M Lee DMX Sun CH Lam JS . The influence of the environment and lifestyle on myopia. J Physiol Anthropol. (2024) 43:7. doi: 10.1186/s40101-024-00354-7, 38297353 PMC10829372

[89] Zhang M Fu Q Zhou S Tan Q Cai G. Meta-analysis of physical activity for the prevention and control of myopia in children and adolescents (in Chinese). Sport Sci Res. (2022) 43:55–64.

[90] Ren YQ Lu ZS. Curricular development of vision training.(in Chinese). J Guangzhou Sport Univ. (2020) 40:95–100. doi: 10.13830/j.cnki.cn44-1129/g8.2020.05.021

[91] Ren DD Yang HM. Effects of orthokeratology combined with visual training and cloud clip on accommodative function. (in Chinese). Int Eye Sci. (2024) 24:1634–9. doi: 10.3980/j.issn.1672-5123.2024.10.21

[92] Wang QJ Li YM Dong YL Xu N. Effects of three optical interventions on myopia progression: a propensity score–matched study (in Chinese). Recent Adv Ophthalmol. (2025) 45:562–5.

[93] Gao YJ Ye CX. Repeated low-level red-light therapy combined with visual training for adolescent myopia. (in Chinese). Int Eye Sci. (2025) 25:1574–9. doi: 10.3980/j.issn.1672-5123.2025.10.05

[94] Liu Y Li W Li L Ren Y Jiang B Chen X . Effects of Chinese acupoint eye exercise on ocular biological parameters. Transl Vis Sci Technol. (2025) 14:20. doi: 10.1167/tvst.14.7.20, 40704879 PMC12309618

[95] Qiao LY Xiang MX Liu AQ. Theoretical basis of intraorbital straight needling for myopia based on the theory of “jingjin imbalance” (in Chinese). Zhongguo Zhen Jiu. (2026). doi: 10.13703/j.0255-2930.20250320-0006

[96] Wang ZJ Zhao S Gu H Jiang H Long QR Chen ZX . Research status of correlation between myopia and accommodative function.(in Chinese). Int Eye Sci. (2024) 24:415–9. doi: 10.3980/j.issn.1672-5123.2024.3.16

[97] Peng X. *Effects of different frequencies of ciliary muscle training in physical education on visual acuity in eighth-grade students* (master’s thesis). Soochow University, Suzhou (2024)(in Chinese)

[98] Wang B Zhao SQ Wang XB. Objective examination and analysis of accommodative function in school-aged children with progressive myopia (in Chinese). Recent Adv Ophthalmol. (2019) 39:433–6.

[99] Yuda K Uozato H Hara N Tetzlaff W Satoru H Horie H . Training regimen involving cyclic induction of pupil constriction during far accommodation improves visual acuity in myopic children. Clin Ophthalmol. (2010) 4:251–60. doi: 10.2147/OPTH.S9249, 20463792 PMC2861931

[100] Chen AM Roberts TL Cotter SA Kulp MT Sinnott LT Borsting EJ . Effectiveness of vergence/accommodative therapy for accommodative dysfunction in children with convergence insufficiency. Ophthalmic Physiol Opt. (2021) 41:21–32. doi: 10.1111/opo.12747, 33119180 PMC10545079

[101] Shyu YL Huang CW Wei SC Yeh SM Liu CT Huang SY . A pilot study investigating immediate effects of vergence training on binocular function and reaction time in youth badminton athletes. Sci Rep. (2025) 15:43180. doi: 10.1038/s41598-025-28743-7, 41345171 PMC12678493

[102] Scheiman M Kulp MT Cotter SA Lawrenson JG Wang L Li T. Interventions for convergence insufficiency: a network meta-analysis. Cochrane Database Syst Rev. (2020) 2020:CD006768. doi: 10.1002/14651858.CD006768.pub3, 33263359 PMC8092638

[103] Yu L Huang TN Qu YM Chen P Wang Y Lv D . Association between myopia progression and visual function in children. (in Chinese). Int Eye Sci. (2024) 24:778–83.

[104] Zhang RX Jiang WJ Bi HS Wen Y. Research progress on the relationship between myopia and accommodative ability.(in Chinese). Recent Adv Ophthalmol. (2020) 40:893–5.

[105] Walline JJ Lindsley K Vedula SS Cotter SA Mutti DO Twelker JD . Interventions to slow progression of myopia in children. Cochrane Database Syst Rev. (2011) 1:CD004916. doi: 10.1002/14651858.CD004916.pub3, 22161388 PMC4270373

[106] Jing T Qiu X Li S Liu J Zhu J Wang X . Intervention efficacy of the revised Mingmu Gong exercise prescription for visual fatigue in university students (in Chinese). Chin Gen Pract. (2026) 29:752–9.

[107] Yang HK Kim DH Kim JH Whangbo TK Hwang JM. Virtual reality head-mounted display game for intermittent exotropia in a randomized controlled trial. Sci Rep. (2025) 15:4317. doi: 10.1038/s41598-024-78088-w, 39910086 PMC11799449

[108] Trbovich AM Zynda AJ Togashi T Burley C Mucha A Collins MW . Randomized controlled trial of Brock string vision therapy for receded near point of convergence following concussion. J Neurotrauma. (2025) 42:1708–18. doi: 10.1177/08977151251359960, 40957852

[109] Rovira-Gay C Argilés M Mestre C . Objective evaluation of fusional vergence after a vision therapy protocol in typical binocular vision. Ophthalmic Physiol Opt. (2025) 45:1173–85. doi: 10.1111/opo.13528, 40411350 PMC12153028

[110] Fazzi E Micheletti S Calza S Merabet L Rossi A Galli J . Early visual training and environmental adaptation for infants with visual impairment. Dev Med Child Neurol. (2021) 63:1180–93. doi: 10.1111/dmcn.14865, 34813110 PMC8518055

[111] Abernethy B Wood JM. Do generalized visual training programmes for sport really work? An experimental investigation. J Sports Sci. (2001) 19:203–22. doi: 10.1080/026404101750095376, 11256825

[112] Zhu MJ He XG Zhu JF. Effect of accommodation optimized training method on improving visual acuity and ocular motor parameters in pre-adolescent myopia.(in Chinese). Recent Adv Ophthalmol. (2012) 32:1034–7. doi: 10.13389/j.cnki.rao.2012.11.013

[113] Sah RP Ramasubramanian V Reed O Meyer D Bradley A Kollbaum PS. Accommodative behavior, hyperopic defocus, and retinal image quality in children viewing electronic displays. Optom Vis Sci. (2020) 97:628–40. doi: 10.1097/OPX.0000000000001543, 32833406

[114] Vivekanand U Lobo SG Dharmappa N. Comparing proximal convergence ratio in myopes. Rom J Ophthalmol. (2021) 65:362–4. doi: 10.22336/rjo.2021.71, 35087977 PMC8764421

[115] Sreenivasan V Irving EL Bobier WR. Effect of heterophoria type and myopia on accommodative and vergence responses during sustained near activity in children. Vis Res. (2012) 57:9–17. doi: 10.1016/j.visres.2012.01.011, 22322001

[116] Chen J Chen YS Wang JJ Yang JLX Xie H Du LL . Randomized controlled clinical trial of solar-spectrum-like LED lighting on retinal blood perfusion in children and adolescents (in Chinese). Chin J Sch Health. (2022) 43:338–40. doi: 10.16835/j.cnki.1000-9817.2022.03.005

[117] Chen X Zuo S Zhang C Sun B Zhang M Jiang D . Interventional study on the effectiveness of eye exercises based on a composite feedback model in school-age children. Risk Manag Healthc Policy. (2024) 17:1787–801. doi: 10.2147/RMHP.S467570, 39007108 PMC11244621

[118] Hu JM Lin X. Zoom-focus training for accommodative myopia. (in Chinese). Recent Adv Ophthalmol. (1993) 3:1–3.

[119] Jiang Y. Benefits of visual-function training for adolescents with accommodative insufficiency. (in Chinese). Electron J Clin Med Lit. (2018) 5:178–9.

[120] Li Y. Effectiveness of accommodative-amplitude training in children with myopia.(in Chinese). Matern Child Health Care China. (2013) 28:4159–19.

[121] Liang JJ Lin P Yao DY Zhang S. Efficacy of visual training for children with visual-function abnormalities and asthenopia.(in Chinese). Int Eye Sci. (2024) 24:1486–90.

[122] Liu HD Ke JL Gong LL Huang Z. Clinical effects of binocular vision and accommodative function training in children with myopia. (in Chinese). J Southwest Med Univ. (2020) 43:492–5.

[123] Pan J. L. *Research on promoting visual health in children and adolescents through physical activity interventions enhancing dynamic visual acuity* (master’s thesis). Soochow University, Suzhou (2021)(in Chinese)

[124] Liu S Wang B Wang GJ Dong J. Effects of three intervention methods on accommodative parameters and refraction in children with myopia. (in Chinese). Int Eye Sci. (2021) 21:1870–4. doi: 10.3980/j.issn.1672-5123.2021.11.07

[125] Mi R Shi J Yang J Chen X Wang D. Effects of three intervention methods on ocular biometric parameters in children with latent myopia.(in Chinese). Int Eye Sci. (2024) 24:1496–501.

[126] Yue H Ren QJ Mei Y. Effects of visual training on myopia progression in adolescents with accommodative lag.(in Chinese). China J Mod Med. (2013) 23:89–91.

[127] Yue J Yue H Ren QJ Zhou Q Huang J. Effects of visual training on positive relative accommodation in adolescents with pseudomyopia (in Chinese). Int Eye Sci. (2014) 14:717–9. doi: 10.3980/j.issn.1672-5123.2014.04.41

[128] Chang MY Morrison DG Binenbaum G Heidary G Trivedi RH Galvin JA . Home- and office-based vergence and accommodative therapies for treatment of convergence insufficiency in children and young adults. Ophthalmology. (2021) 128:1756–65. doi: 10.1016/j.ophtha.2021.05.017, 34172337

[129] Horie H Hisahara S Horie H Nakajima S Hara N Watanabe K . (2009). To keep the object’s retinal projection size constant is essential for induction of a pupillary constriction during far accommodation. (conference abstract) Meeting of the association for research in vision and ophthalmology, May 3–7, 2009, Fort Lauderdale, FL, S3923.

[130] Jiang BC Bussa S Tea YC Seger K. Optimal dioptric value of near addition lenses intended to slow myopic progression. Optom Vis Sci. (2008) 85:1100–5. doi: 10.1097/OPX.0b013e31818b9f47, 18981925

[131] Le RR Zheng ZL Song JL Lü F. Differences in accommodative microfluctuations between emmetropic and myopic adolescents and their relationship with wavefront aberrations. (in Chinese). Chin J Exp Ophthalmol. (2015) 33:745–50.

[132] Luo J Tao LJ Qi ZY Guo Y Dong CL. Study on AC/a ratio in myopia children. (in Chinese). Int Eye Sci. (2010) 10:76–7. doi: 10.3969/j.issn.1672-5123.2010.01.023

[133] Xu D Chen X Yu Y Wang Y Wang H Lin L. Accommodation response fluctuation in myopia during sustained near work. (in Chinese). Zhonghua Yan Ke Za Zhi. (2009) 45:50–5. 19484931

[134] Wang HY Zhao KX. The effects of myopic anisometropia on binocular vision function.(in Chinese). Chin J Exp Ophthalmol. (2013) 31:559–63. doi: 10.3760/cma.j.issn.2095-0160.2013.06.009

[135] Wang B Ciuffreda KJ Vasudevan B. Effect of blur adaptation on blur sensitivity in myopes. Vis Res. (2006) 46:3634–41. doi: 10.1016/j.visres.2006.03.015, 16697436

[136] Yang YG. The meaning of measuring gradient AC/a in preventing and correcting adolescent myopia.(in Chinese). Int Eye Sci. (2013) 13:1291–2. doi: 10.3980/j.issn.1672-5123.2013.06.72

[137] Wang M Zhu G Li Y Li P Shi H Jiang L . The impact of physical exercise with additional visual tasks on UDVA and accommodation sensitivity in children: the mediating role of kinetic visual acuity. Front Public Health. (2024) 12:1467651. doi: 10.3389/fpubh.2024.1467651, 39712315 PMC11662335

[138] Olawade DB Weerasinghe K Mathugamage MDDE Odetayo A Aderinto N Teke J . Enhancing ophthalmic diagnosis and treatment with artificial intelligence. Medicina. (2025) 61:433. doi: 10.3390/medicina61030433 40142244 PMC11943519

