# Supplementary material for: Exercise and visual training interventions for myopia in children and adolescents: a systematic review with a focus on near–far accommodation
Source: Front Public Health. 2026 Aug 12;14:1889393. doi: 10.3389/fpubh.2026.1889393 (PMC13506763; doi:10.3389/fpubh.2026.1889393)
Supplement: Supplementary file 1 [file Data_Sheet_1.PDF]

Supplementary Material

1 Supplementary Figures and Tables

1.1 Supplementary Figures

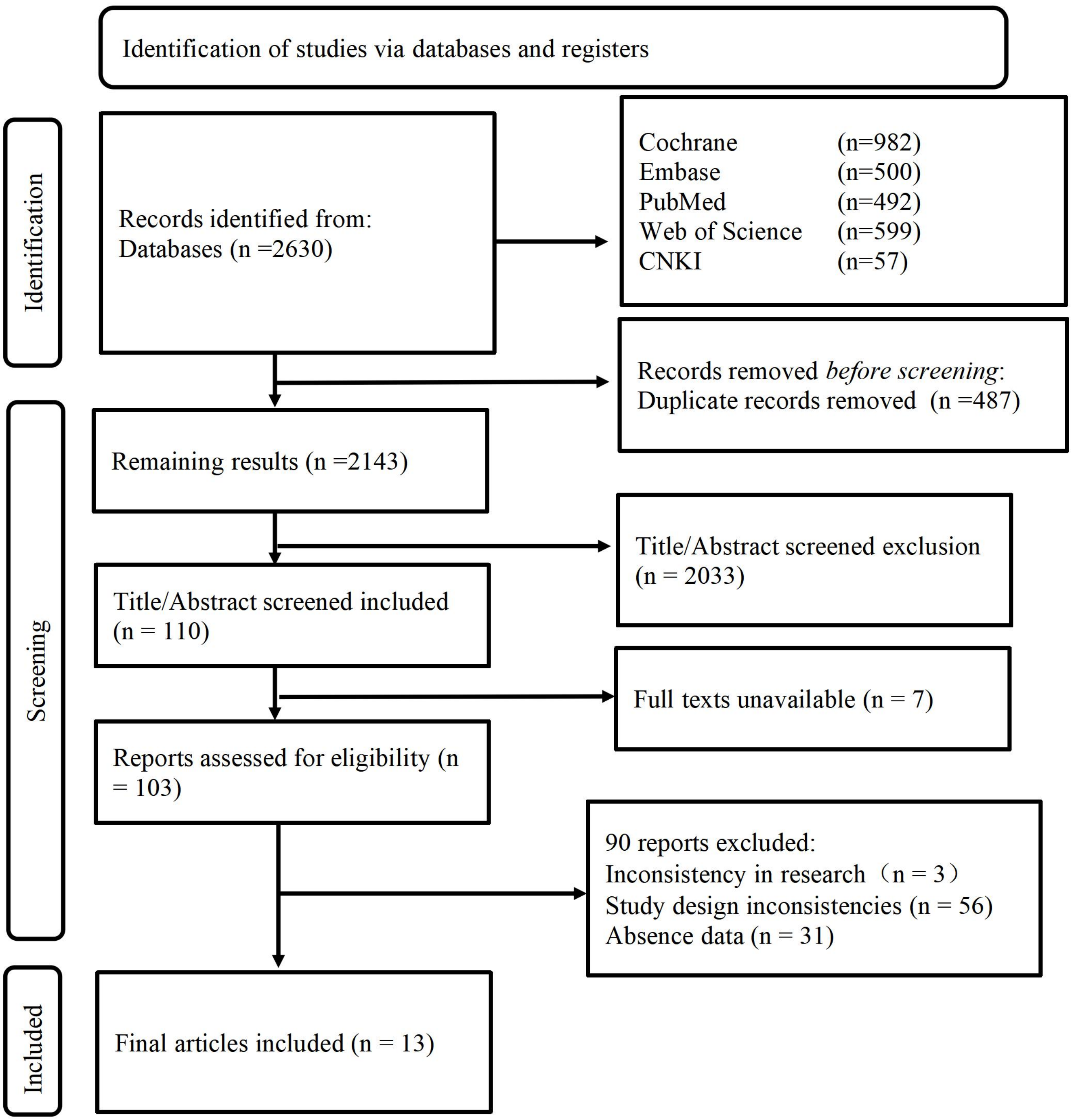

Figure 1. PRISMA Flow Diagram of the Study Selection Process

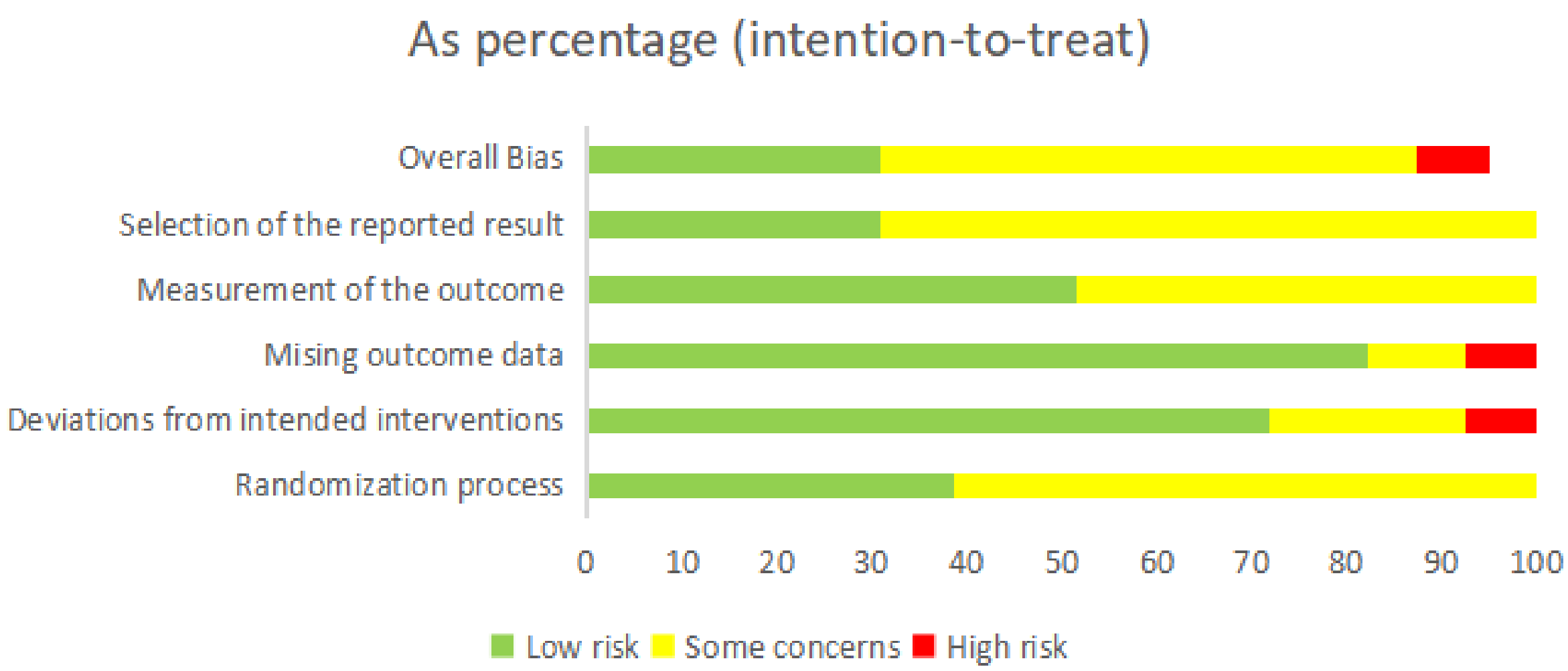

Figure 2. Risk-of-bias plot

Note: Figure 2 presents only the RoB 2 assessment results for the 11 individually randomized controlled trials; the results for the cluster-randomized study and the non-randomized study are presented in Table 2.

1.2 Supplementary Tables

Table 1. Inclusion Criteria Based on the PICOS Framework

PICOS	Details
Population	School-aged children and adolescents, both male and female, aged 6–18 years.
Intervention	Any form of accommodative visual training, near–far accommodative training, or accommodation-related visual intervention embedded in physical activity.
Comparator	Regular study routines, daily activities, regular physical education or ordinary sports activities, or no-intervention control.
Outcomes	Visual performance indicators, including uncorrected visual acuity, binocular visual acuity, results measured separately for the right and left eyes, and kinetic visual acuity; ocular structural or refractive indicators, including axial length, spherical equivalent refraction, refractive error, and corneal curvature; accommodative function indicators, including accommodative lag, accommodative facility, accommodative amplitude, and accommodative accuracy; and other visual health-related indicators.
Study design	Randomized controlled trials and non-randomized interventional studies were included, including quasi-experimental studies in which intervention and control groups were assigned by the investigators. Studies were required to include a comparable control group and to measure the primary outcomes consistently before and after the intervention. Single-group pre–post studies without a control group were not included in the primary evidence synthesis.

Table 2. Risk-of-Bias Assessment

No.	Study	Study design	Assessment tool	Outcomes assessed	Overall risk of bias
1	Xu et al., 2024	Individually randomized controlled trial (RCT)	RoB 2 (individual randomized parallel-group trial)	AL, mCT, UCVA, NVA	Some concerns
2	Xie et al., 2024	Individually randomized controlled trial (RCT)	RoB 2 (individual randomized parallel-group trial)	AL, SER, AE	Low
3	Zheng et al., 2022	Individually randomized controlled trial (RCT)	RoB 2 (individual randomized parallel-group trial)	UCVA (left eye, right eye, and binocular)	High
4	Yin et al., 2022	Individually randomized controlled trial (RCT)	RoB 2 (individual randomized parallel-group trial)	KVA, UDVA	Low
5	Zhou et al., 2023	Individually randomized controlled trial (RCT)	RoB 2 (individual randomized parallel-group trial)	KVA, UDVA, AL, AF	Some concerns
6	Han et al., 2023	Individually randomized controlled trial (RCT)	RoB 2 (individual randomized parallel-group trial)	UCVA (left and right eyes), detection rate of poor vision	Some concerns
7	Lin et al., 2025	Individually randomized controlled trial (RCT)	RoB 2 (individual randomized parallel-group	SER/SE, AL, K, AL/CR, AMP, NRA, PRA, AF	Some concerns

No.	Study	Study design	Assessment tool	Outcomes assessed	Overall risk of bias
			trial)		
8	Wang et al., 2023	Individually randomized controlled trial (RCT)	RoB 2 (individual randomized parallel-group trial)	UCVA, refractive error, visual fatigue	Some concerns
9	Xie et al., 2013	Individually randomized controlled trial (RCT)	RoB 2 (individual randomized parallel-group trial)	Myopic refractive error/SE, AL	Low
10	Allen et al., 2009	Individually randomized controlled trial (RCT)	RoB 2 (individual randomized parallel-group trial)	LoA, distance AF, near AF, PRT, NRT	Some concerns
11	Hu et al., 2025	Individually randomized controlled trial (RCT)	RoB 2 (individual randomized parallel-group trial)	ChT (0–1 mm and 1–3 mm), monocular AF	Some concerns
12	Zhao et al., 2008	Cluster-randomized controlled trial (class-level randomization)	RoB 2 CRT	Change in cycloplegic refractive classification after 4 months; change in UCVA after 4 months	High
13	Jin et al., 2021	Non-randomized comparative intervention study	ROBINS-I V2	Change in UCVA after 16 weeks, KVA	Serious

Note: Individually randomized controlled trials were assessed using RoB 2; the cluster-randomized study was assessed using RoB 2 CRT; and the non-randomized comparative intervention study was assessed using ROBINS-I V2. The assessment domains and risk judgment levels are not fully identical across these tools.

Table 3. Characteristics of Included Studies

Study	Sample (T/C)	Age	Intervention (T/C)	Duration (weeks)	Frequency (sessions/week)	Session duration (min/session)	Outcome measures	Main findings
Xu et al., 2024	Students (30/30)	T: 10.53 ± 1.57; C: 10.67 ± 1.30	SVS + VR-based visual training / SVS	12	7	20	UDVA, NVA, AL, mCT	AL: T baseline, 24.66 ± 0.64 mm; 3-month Δ, 0.063 ± 0.060 mm, between-group P = 0.037; 6-month Δ, 0.166 ± 0.109 mm, P = 0.172. mCT: T baseline, 290.77 ± 48.25 μm; 3-month Δ, +22.63 ± 36.17 μm, P = 0.005; 6-month P = 0.145. UDVA and NVA improved at 3 months (P = 0.000 and P = 0.034, respectively).
Xie et al., 2024	Students (131/132)	6–18 years; T: 11.04 ± 1.96; C: 10.58 ± 1.96	Naked-eye three-dimensional vision training / normal daily life	24	7	20	AL, SER	AL: T baseline, 24.35 ± 0.83 mm; 6-month Δ, 0.176 mm (95% CI: 0.155 to 0.196); between-group difference, −0.056 mm (95% CI: −0.085 to −0.027). SER: T baseline, −2.08 (1.12) D; 6-month Δ, −0.251 D (95% CI: −0.311 to −0.191).
Zheng et al., 2022	Students (26/27)	6–18 years	Eye-protection exercises + table tennis / regular study and daily life	24	7; 3	20; 45	UDVA-L, UDVA-R, BVA	UDVA-L: 0.565 ± 0.042 to 0.912 ± 0.041 at 3 months (Δ +0.347); 0.908 ± 0.042 at 6 months (Δ +0.343), both P < 0.01. UDVA-R: 0.539 ± 0.042 to 0.881 ± 0.047 at 3 months (Δ +0.342); 0.877 ± 0.046 at 6 months (Δ +0.338), both P < 0.01. BVA: 0.704 ± 0.048 to 1.062 ± 0.046 at 3 months; 1.054 ± 0.045 at 6 months, both P < 0.01.
Yin et al., 2022	Students (44/40/43/33)	10–11 years	Physical activity-embedded near–far accommodative training (15-, 30-, and 60-session	16	3	40	KVA, UDVA	KVA: 30-session group, 0.268 ± 0.214 to 0.486 ± 0.327 (Δ +0.218); 60-session group, 0.333 ± 0.304 to 0.424 ± 0.324 (Δ +0.091); 15-session group, P > 0.05. UDVA: 30-session group, 4.775 ± 0.306 to

Study	Sample (T/C)	Age	Intervention (T/C)	Duration (weeks)	Frequency (sessions/week)	Session duration (min/session)	Outcome measures	Main findings
			groups) / regular physical activity					4.937 ± 0.269 (Δ +0.162, P < 0.05); 60-session group, 4.807 ± 0.335 to 4.958 ± 0.280 (Δ +0.151, P < 0.05).
Zhou et al., 2023	Students (39/38/38/38)	10–11 years	Ciliary muscle training embedded in PE class with different visual target presentation durations (1 s/3 s/5 s) / same physical activity	32	3	40	KVA, UDVA, AL, AF	In the 1-s, 3-s, and 5-s groups, KVA increased by +0.10, +0.12, and +0.09, respectively; UDVA increased by +0.11, +0.14, and +0.11, respectively; AL increased by +0.20, +0.20, and +0.22 mm, respectively; and AF increased by +0.22, +0.26, and +0.16 cpm, respectively.
Han et al., 2023	Students (75/127)	9.5 years	Regular classes + tennis lessons / extracurricular activities and regular PE class	40	2; 4	40; 30	UDVA-L, UDVA-R, detection rate of poor vision	Boys' UDVA-L/R: T baseline, 5.06 ± 0.16 / 5.08 ± 0.11; 10 months, 5.00 ± 0.13 / 5.00 ± 0.12 (Δ −0.06 / −0.08). In the third assessment, T was higher than C (P = 0.02). Girls' UDVA-L/R: T baseline, 5.05 ± 0.16 / 5.05 ± 0.15; 10 months, 4.97 ± 0.13 / 4.97 ± 0.14 (Δ −0.08 / −0.08). In the third assessment, T was higher than C (P < 0.01). The detection rate of poor vision was lower than that in the control group (P < 0.01).
Lin et al., 2025	Students (12/12/12)	Table tennis: 7.4 ± 1.43; badminton: 7.9 ± 1.10; C: 8.20 ±	Table tennis or badminton / regular PE games	24	3	90	SER, AL, AF	Table tennis group: left/right SER Δ −0.18 / −0.10 D (both P > 0.05); left/right AL Δ +0.54 / +0.65 mm (both P < 0.05); AF improved significantly in both eyes (P < 0.05). Badminton group: left-eye SER Δ −0.04 D (P < 0.05), right-eye SER Δ −0.15 D (P > 0.05); left/right AL Δ +0.68 / +0.52

Study	Sample (T/C)	Age	Intervention (T/C)	Duration (weeks)	Frequency (sessions/week)	Session duration (min/session)	Outcome measures	Main findings
		1.75						mm (both P < 0.05).
Wang et al., 2023	Students (60/60)	10–11 years	Orienteering visual training / regular study and daily life	10	3	45	UDVA, SE	UDVA: 4.66 ± 0.05 to 4.73 ± 0.64 (Δ +0.07, P < 0.01). SE: −3.35 ± 0.92 to −2.99 ± 0.25 D (Δ +0.36, P < 0.01).
Xie et al., 2013	Students (53/53)	T: 10.61 ± 1.77; C: 10.83 ± 1.69	Near-use convex-lens prism + physiological visual eye exercises / near-use convex-lens prism	104	14	3	SER, AL	SER: −1.17 ± 0.47 to −2.05 ± 0.81 D at 2 years (Δ −0.81 D, P < 0.05; between-group P = 0.0001). AL: 23.91 ± 0.66 to 24.21 ± 0.36 mm (Δ +0.23 mm, P < 0.05; between-group P = 0.023).
Zhao et al., 2008	Students (39/37)	T: 10.4; C: 10.3	SU-001 ciliary muscle training / no training	16	14	15	UDVA, SER	UDVA: 0.71 ± 0.36 to 1.20 ± 0.56 at 4 months (Δ +0.49, P < 0.01).
Jin et al., 2021	Students (39/32; no blank control group)	NR; Grade 4 students	Cheerleading-based dynamic visual acuity training / football-based dynamic visual acuity training	16	3	40	KVA, UDVA	Cheerleading group: KVA, 0.338 ± 0.240 to 0.497 ± 0.249 (Δ +0.159); UDVA, 4.526 ± 0.245 to 4.741 ± 0.241 (Δ +0.215). Football group: KVA, 0.315 ± 0.228 to 0.549 ± 0.203 (Δ +0.234); UDVA, 4.491 ± 0.135 to 4.600 ± 0.134 (Δ +0.109).
Allen et al., 2009	Myopic participants (n = 93)	16.7 ± 2.1 years; only participants younger than 18	Spherical-aberration-modified contact lenses and/or visual training /	6	7	18	LoA, DAF, NAF	LoA decreased in the contact lens treatment group (F = 11.23, P = 0.001), whereas it increased in the contact lens control group (P = 0.029). No significant effect was observed in the visual training group. NAF:

Study	Sample (T/C)	Age	Intervention (T/C)	Duration (weeks)	Frequency (sessions/week)	Session duration (min/session)	Outcome measures	Main findings
		years were considered	control contact lenses and/or no training					VT treatment group, 5.19 ± 2.33 to 7.27 ± 2.05 cpm (Δ +2.08, P < 0.001). DAF improved in both groups, but no between-group difference was observed (P = 0.158).
Hu et al., 2025	Students (11/9)	T: 9.36 ± 1.75; C: 8.44 ± 1.81	AR-based visual training / rest	Single session	1	30	ChT, AF, SER	ChT in the 0–1 mm zone: 332.85 ± 79.00 to 324.11 ± 73.52 μm (Δ −8.74 μm, 95% CI: −13.81 to −3.68, P = 0.007). ChT in the 1–3 mm zone: 327.30 ± 74.54 to 318.60 ± 68.60 μm (P = 0.018). Monocular AF: AR group, 5.05 ± 1.38 to 8.05 ± 1.81 cpm (Δ +3.00, P = 0.004); no significant change was observed in the control group (P = 0.367). Binocular AF showed no significant change (P = 0.127).

Note. Δ represents the change value. T = treatment/intervention group; C = control group; SD = standard deviation; CI = confidence interval; MD = mean difference; P = probability value; D = diopter; cpm = cycles per minute; SVS = single-vision spectacles; VR = virtual reality; AR = augmented reality. UDVA = uncorrected distance visual acuity; NVA = near visual acuity; BVA = binocular visual acuity; KVA = kinetic visual acuity; AL = axial length; SER = spherical equivalent refraction; mCT = macular choroidal thickness; ChT = choroidal thickness; CC = corneal curvature. AF = accommodative facility; AMP = amplitude of accommodation; LoA = lag of accommodation; DAF = distance accommodative facility; NAF = near accommodative facility; PRA = positive relative accommodation; NRA = negative relative accommodation.

Table 4. Full-text excluded studies and reasons for exclusion

Study	Reasonforexclusion
He et al. (2022)	Did not meet review criteria as the intervention was outdoor time/light exposure rather than near-far

Study	Reasonforexclusion
	accommodative or visual training.
Li et al. (2018)	Did not meet review criteria as the intervention focused on outdoor exposure rather than near-far accommodative training.
Ying et al. (2024)	Not eligible as this was an umbrella review of systematic reviews and meta-analyses.
Yu et al. (2023)	Not eligible as this was a systematic review and meta-analysis of risk factors.
Morgan et al. (2018)	Not eligible as this was a narrative review on myopia aetiology and prevention.
Dhakal et al. (2022)	Not eligible as this was an overview of systematic reviews.
Xiong et al. (2017)	Not eligible as this was a systematic review and meta-analysis of outdoor activities.
Cheng and Zhang (2025)	Not eligible as this was a systematic review and network meta-analysis.
Li et al. (2019)	Not eligible as this was a narrative review.
Xie et al. (2025)	Not eligible as this was a cross-sectional prediction-model study.
Zhang et al. (2025)	Not eligible as this was a cross-sectional survey on myopia prevalence and influencing factors.
Xu et al. (2025)	Not eligible as this was a cross-sectional survey on myopia status and influencing factors.
Wang et al. (2022)	Did not meet review criteria as participants were patients with primary open-angle glaucoma.

Study	Reasonforexclusion
Wang (2023)	Not eligible as this was an observational study without an intervention.
Read et al. (2015)	Not eligible as this was a longitudinal observational study of light exposure.
Fan et al. (2023)	Did not meet review criteria as the intervention was a general badminton exercise prescription without clearly defined near-far accommodative training.
Wu (2024)	Not eligible as this was a master's thesis rather than a peer-reviewed journal article.
Pan (2021)	Not eligible as this was a master's thesis rather than a peer-reviewed journal article.
Zhang (2021)	Not eligible as this was a master's thesis rather than a peer-reviewed journal article.
Nie (2021)	Not eligible as this was a master's thesis rather than a peer-reviewed journal article.
Zhou (2021)	Not eligible as this was a master's thesis rather than a peer-reviewed journal article.
Yan (2021)	Not eligible as this was a master's thesis rather than a peer-reviewed journal article.
Chen (2024)	Not eligible as this was a master's thesis rather than a peer-reviewed journal article.
Xue et al. (2023)	Not eligible as this was a cross-sectional study on gait kinematics, not an intervention study.
Huang (2021)	Not eligible as this was a master's thesis rather than a peer-reviewed journal article.
Xie (2021)	Not eligible as this was a master's thesis rather than a peer-reviewed journal article.

Study	Reasonforexclusion
Song et al. (2002)	Not eligible as this was a comparative observational study rather than a controlled intervention study.
Chen et al. (2018)	Not eligible as this was a cross-sectional study on dynamic visual acuity and table-tennis training experience.
Jouira et al. (2024)	Did not meet review criteria as the outcome was postural balance rather than myopia-related visual function.
Liu et al. (2024)	Not eligible as this was a network meta-analysis, not a primary intervention study.
Tao et al. (2019)	Not eligible as this was an expert recommendation rather than a primary intervention study.
Chen et al. (2025)	Not eligible as this was a prospective cohort study rather than an intervention study.
Lundberg et al. (2018)	Not eligible as this was an observational cohort study without an intervention.
Li et al. (2021)	Did not meet review criteria as the intervention was acute physical exercise rather than near-far accommodative or visual training.
Mei et al. (2024)	Not eligible as this was a systematic review and meta-analysis.
Al-Mohtaseb et al. (2021)	Not eligible as this was a review on dry eye disease and digital screen use.
Xu et al. (2023)	Not eligible as this was a cross-sectional study on sleep-wake schedules and self-reported myopia.
Wang et al. (2016)	Did not meet review criteria as participants were medical students and the outcome was visual fatigue rather than myopia-related visual function.

Study	Reasonforexclusion
Yang et al. (2016)	Not eligible as this was a cross-sectional correlation study on physical activity and myopia.
O'Donoghue et al. (2015)	Not eligible as this was an observational risk-factor study rather than an intervention study.
Liao et al. (2023)	Did not meet review criteria as the intervention was outdoor illumination and general exercise rather than near-far accommodative or visual training.
Ma et al. (2024)	Not eligible as this was a cross-sectional study on physical activity and myopia prevalence.
Lu et al. (2022)	Did not meet review criteria as the outcome was short-term eye strain relief rather than myopia-related visual function.
Biswas et al. (2024)	Not eligible as this was a narrative review on environmental and lifestyle factors related to myopia.
Zhang et al. (2022)	Did not meet review criteria as the outcome focused on physical and mental health rather than myopia-related visual function.
Ren and Lu (2020)	Not eligible as this was a theoretical study on visual training curriculum rather than a primary intervention study.
Ren and Yang (2024)	Did not meet review criteria as visual training was combined with orthokeratology and cloud-clip monitoring.
Wang et al. (2025)	Did not meet review criteria as the intervention was optical lens treatment rather than near-far accommodative or visual training.
Gao and Ye (2025)	Did not meet review criteria as visual training was combined with orthokeratology and repeated low-energy red-light therapy.

Study	Reasonforexclusion
Liu et al. (2025)	Not eligible as this was a review article rather than a primary intervention study.
Qiao et al. (2026)	Not eligible as this was a theoretical article on acupuncture rather than a primary intervention study.
Wang (2024)	Not eligible as this was a master's thesis rather than a peer-reviewed journal article.
Peng (2024)	Not eligible as this was a master's thesis rather than a peer-reviewed journal article.
Wang et al. (2019)	Not eligible as this was an observational study of accommodative function rather than an intervention study.
Yuda et al. (2010)	Did not meet review criteria as no eligible comparable control group was included.
Chen et al. (2021)	Did not meet review criteria as participants had convergence insufficiency with accommodative dysfunction rather than myopia-related outcomes.
Shyu et al. (2025)	Did not meet review criteria as this was an immediate-effect pilot study in youth badminton athletes.
Scheiman et al. (2020)	Not eligible as this was a network meta-analysis rather than a primary intervention study.
Yu et al. (2024)	Not eligible as this was a narrative review on visual function and myopia progression.
Wang et al. (2024)	Not eligible as this was a narrative review on myopia and accommodative function.
Zhang et al. (2020)	Not eligible as this was a narrative review on the relationship between myopia and accommodation.

Study	Reasonforexclusion
Walline et al. (2011)	Not eligible as this was a systematic review rather than a primary intervention study.
Jing et al. (2026)	Did not meet review criteria as participants had intermittent exotropia rather than myopia.
Yang et al. (2025)	Did not meet review criteria as participants had intermittent exotropia rather than myopia.
Trbovich et al. (2025)	Did not meet review criteria as participants had concussion-related receded near point of convergence.
Rovira-Gay et al. (2025)	Did not meet review criteria as participants were adults with typical binocular vision rather than myopic children or adolescents.
Fazzi et al. (2021)	Did not meet review criteria as participants were infants with visual impairment rather than school-aged children with myopia.
Abernethy and Wood (2001)	Did not meet review criteria as this was a sports vision training study without myopia-related outcomes.
Sun et al. (2018)	Full text unavailable.
Zhu et al. (2018)	Full text unavailable.
Liu et al.（2024）	Full text unavailable.
Weerasingheet al.（2025）	Full text unavailable.
Sah et al.（2020）	Full text unavailable.

Study	Reasonforexclusion
Vivekanand et al.（2021）	Full text unavailable.
Sreenivasan et al.（2012）	Full text unavailable.
Chen et al. (2022)	Intervention was light exposure rather than accommodative or visual training.
Chen et al. (2024)	Intervention was traditional Chinese medicine therapy rather than accommodative or visual training.
Hu and Lin (1993)	Insufficient controlled intervention data.
Jiang (2018)	Not a controlled intervention study.
Li (2013)	No comparable control group.
Li (2019)	Master’s thesis, not a peer-reviewed journal article.
Liang et al. (2024)	No comparable control group and outcomes were not focused on myopia control.
Liu et al. (2020)	Combined multiple interventions and the effect of accommodative training could not be isolated.
Liu et al. (2021)	Intervention was not accommodative or visual training.
Mi et al. (2024)	Included pharmacological treatment and did not match the target intervention.
Yue et al. (2013)	Intervention did not match the predefined visual training criteria.

Study	Reasonforexclusion
Yue et al. (2014)	Participants had pseudomyopia rather than myopia.
Chang et al. (2021)	Outcomes were not related to myopia.
Horie et al.(2010)	No eligible control group.
Rongbin Yin et al. (2022)	Duplicate or related report of an included study.
Jiang (2008)	Correlation study; not an intervention.
Le et al. (2015)	Observational study; not an intervention.
Luo et al. (2010)	Observational study; not an intervention.
Xu et al. (2009)	Case-control study; not an intervention.
Wang et al. (2013)	Observational study; not an intervention.
Wang et al. (2006)	Observational study; not an intervention.
Yang (2013)	Clinical report; not an intervention.

1.3 Supplementary File 1. Search strategies

Boolean Search Formula

1.3.1 PubMed

#1 Myopia block:
("Myopia"[Mesh] OR myopia*[tiab] OR myopi*[tiab] OR "near-sight*"[tiab] OR "short-sight*"[tiab] OR nearsighted*[tiab] OR shortsighted*[tiab] OR "refractive error*"[tiab] OR "spherical equivalent"[tiab] OR "axial length"[tiab])
#2 Children/adolescents:
("Child"[Mesh] OR "Adolescent"[Mesh] OR child*[tiab] OR adolescent*[tiab] OR teen*[tiab] OR schoolchild*[tiab] OR student*[tiab] OR pupil*[tiab] OR pediatric*[tiab] OR paediatric*[tiab])
#3 Visual/accommodation training:
("Accommodation, Ocular"[Mesh] OR accommodat*[tiab] OR "accommodative lag"[tiab] OR "accommodative facility"[tiab] OR "accommodative response"[tiab] OR "ciliary muscle"[tiab] OR "vision therap*"[tiab] OR "visual training"[tiab] OR "eye exercise*"[tiab] OR orthoptic*[tiab] OR "vergence accommodative therap*"[tiab] OR "near-far"[tiab] OR "far-near"[tiab] OR "distance-near"[tiab] OR "virtual reality visual training"[tiab] OR VRVT[tiab])
#4 Exercise/outdoor intervention:
("Exercise"[Mesh] OR "Exercise Therapy"[Mesh] OR "Motor Activity"[Mesh] OR "Sports"[Mesh] OR "Sunlight"[Mesh] OR "outdoor activity"[tiab] OR "outdoor time"[tiab] OR "outdoor intervention*"[tiab] OR "physical activit*"[tiab] OR exercise[tiab] OR sport*[tiab] OR "school recess"[tiab] OR "outdoor light"[tiab] OR sunlight[tiab] OR daylight[tiab])
#5 Study design filter:
("Randomized Controlled Trial"[Publication Type] OR "Controlled Clinical Trial"[Publication Type] OR randomized[tiab] OR randomised[tiab] OR trial[tiab] OR intervention*[tiab] OR "controlled trial"[tiab] OR quasi-experiment*[tiab])
Final: #1 AND #2 AND (#3 OR #4) AND #5 NOT (animals[mh] NOT humans[mh])

1.3.2 Cochrane Library (CENTRAL)

#1 MeSH descriptor: [Myopia] explode all trees
#2 (myopia OR myopi OR nearsighted OR shortsighted OR (refractive error*) OR (spherical equivalent) OR (axial length)):ti,ab,kw
#3 MeSH descriptor: [Child] explode all trees OR MeSH descriptor: [Adolescent] explode all trees
#4 (child OR adolescent OR teen OR schoolchild OR student* OR pupil* OR pediatric* OR paediatric*):ti,ab,kw
#5 MeSH descriptor: [Accommodation, Ocular] explode all trees
#6 (accommodat* OR "accommodative lag" OR "accommodative facility" OR "visual training" OR "vision therap*" OR "eye exercise*" OR orthoptic* OR "near-far" OR "far-near" OR "virtual reality visual training" OR VRVT):ti,ab,kw
#7 MeSH descriptor: [Exercise] explode all trees OR MeSH descriptor: [Exercise Therapy] explode all trees OR MeSH descriptor: [Motor Activity] explode all trees OR MeSH descriptor: [Sports] explode all trees OR MeSH descriptor: [Sunlight] explode all trees
#8 ("outdoor activity" OR "outdoor time" OR "outdoor intervention*" OR "physical activit*" OR exercise OR sport* OR "school recess" OR sunlight OR daylight):ti,ab,kw
#9 (#1 OR #2) AND (#3 OR #4) AND ((#5 OR #6) OR (#7 OR #8))

1.3.3 Embase (Ovid/Elsevier)

#1 'myopia'/exp OR (myopia* OR myopi* OR nearsighted* OR shortsighted* OR 'refractive error*' OR 'spherical equivalent' OR 'axial length'):ti,ab,kw

#2 'child'/exp OR 'adolescent'/exp OR (child* OR adolescen* OR teen* OR schoolchild* OR student* OR pupil* OR pediatric* OR paediatric* OR 'school age*' OR 'school-age*'):ti,ab,kw

#3 'accommodation'/exp OR 'orthoptics'/exp OR (accommodat* OR 'accommodative lag' OR 'accommodative facility' OR 'accommodative response' OR 'ciliary muscle*' OR 'visual training' OR 'vision therap*' OR 'eye exercise*' OR orthoptic* OR 'near far' OR 'far near' OR 'near-far' OR 'far-near' OR 'virtual reality visual training' OR VRVT):ti,ab,kw

#4 'exercise'/exp OR 'exercise therapy'/exp OR 'motor activity'/exp OR 'sport'/exp OR 'sunlight'/exp OR ('outdoor activit*' OR 'outdoor time' OR 'outdoor intervention*' OR 'outdoor light' OR 'physical activit*' OR exercise* OR sport* OR 'school recess' OR sunlight OR daylight):ti,ab,kw

#5 'randomized controlled trial'/exp OR 'controlled clinical trial'/de OR random*:ti,ab,kw OR randomization:ti,ab,kw OR trial*:ti,ab,kw OR intervention*:ti,ab,kw OR 'controlled trial*':ti,ab,kw OR 'quasi experiment*':ti,ab,kw OR 'quasi-experiment*':ti,ab,kw

#6 #1 AND #2 AND (#3 OR #4) AND #5

#7 #6 AND [humans]/lim

1.3.4 Web of Science Core Collection

TS=((myopia* OR myopi* OR nearsighted* OR shortsighted* OR "refractive error*" OR "spherical equivalent" OR "axial length") AND (child* OR adolescent* OR teen* OR schoolchild* OR student* OR pupil* OR pediatric* OR paediatric*) AND ((accommodat* NEAR/3 (train* OR therap* OR lag OR facility OR response OR dysfunction)) OR "vision therapy" OR "visual training" OR "eye exercise*" OR orthoptic* OR "near-far" OR "far-near" OR "ciliary muscle" OR "virtual reality visual training" OR VRVT OR (outdoor NEAR/3 (activit* OR time OR intervention* OR exposure OR light)) OR "physical activit*" OR exercise OR sport* OR "school recess" OR sunlight OR daylight OR "light intensity") AND (random* OR trial* OR intervention* OR "controlled trial" OR "cluster randomized" OR "cluster randomised" OR quasi-experiment*))

1.3.5 Alternative CNKI strategy without study-design filter

Database: CNKI (China National Knowledge Infrastructure)
Collections: CNKI Journal (CJFD), Master's & Doctoral Dissertations, Conference Proceedings (as available)
Field: Subject/Theme (主题)
Time span: Inception to May 1, 2026 (no start-date restriction)
Planned final run date: May 1, 2026

#Step	Search Terms and Boolean Logic
#1	SU=近视 OR 屈光不正 OR 视力 OR 裸眼视力 OR 动态视力 OR 运动视力 OR 视觉健康 OR 眼轴 OR 眼轴长度 OR 等效球镜 OR 球镜等效 OR 屈光度 OR 屈光进展 OR 调节功能 OR 调节灵敏度 OR 调节幅度 OR 调节滞后 OR 睫状肌 OR 脉络膜厚度
#2	SU=儿童 OR 青少年 OR 学生 OR 小学生 OR 中学生 OR 学龄儿童 OR 少儿
#3	SU=调节 OR 眼调节 OR 远近调节 OR 近远调节 OR 调节训练 OR 睫状肌训练 OR 视觉训练 OR 视功能训练 OR 视觉治疗 OR 眼部训练 OR 眼操 OR 眼保健操 OR 视觉生理性眼操 OR 动态视觉 OR 运动视觉 OR 视觉追踪 OR 动态视觉追踪 OR 注视转换 OR 聚焦转换 OR 再聚焦 OR 远近目标 OR 近远目标 OR 裸眼 3D OR 3D 视觉训练 OR 虚拟现实 OR 增强现实 OR VR OR AR
#4	SU=体育活动 OR 体育锻炼 OR 运动干预 OR 运动训练 OR 身体活动 OR 户外活动 OR 体育课
#5	#3 OR #4
#6	#1 AND #2 AND #5

Records retrieved from CNKI: 57 records.

The final CNKI query was executed using CNKI Advanced Search. Any database-specific syntax adjustments were recorded in the search log.
